# Role of miRNAs as biomarkers of COVID-19: a scoping review of the status and future directions for research in this field

**DOI:** 10.2217/bmm-2021-0348

**Published:** 2021-11-17

**Authors:** Marília B Visacri, Aline de S Nicoletti, Eder de C Pincinato, Pía Loren, Nicolás Saavedra, Kathleen Saavedra, Luis A Salazar, Patricia Moriel

**Affiliations:** ^1^Department of Pharmacology, School of Medical Sciences, University of Campinas, Campinas, 13083-887, Brazil; ^2^Department of Clinical Pathology, School of Medical Sciences, University of Campinas, Campinas, 13083-887, Brazil; ^3^Center of Molecular Biology & Pharmacogenetics, Scientific & Technological Bioresource Nucleus, Universidad de La Frontera, Temuco, 4811230, Chile; ^4^Faculty of Pharmaceutical Sciences, University of Campinas, Campinas, 13083-871, Brazil

**Keywords:** biomarkers, COVID-19, epigenomics, miRNAs, review, SARS-CoV-2

## Abstract

**Aim:** miRNAs are potential biomarkers of several diseases. This review aimed to identify the miRNAs that could serve as biomarkers of COVID-19. **Materials & methods:** A literature search of nine databases was carried out for studies published before 13 June 2021 that described dysregulated miRNAs in cells or animals infected by SARS-CoV-2 or in patients with COVID-19. Two independent reviewers selected the studies and extracted data; disagreements were resolved by a third reviewer. **Results:** Twenty studies were included in this scoping review; results suggested that miR-21-5p, miR-146a, miR-126-3p, miR-144 and miR-155 are the most important dysregulated miRNAs that could serve as biomarkers for diagnosing and indicating the severity of COVID-19. miRNAs appear to play key roles in viral replication, proliferation of infected cells, immune response, inflammation and cardiovascular dysfunction. **Conclusion:** This review provides insights into the role of miRNAs as biomarkers in COVID-19 and the current status and future directions for research in this field.

SARS-CoV-2 is a novel betacoronavirus that has been identified as the infectious agent responsible for COVID-19 [1]. After the initial outbreak in Wuhan, China, COVID-19 spread worldwide; the COVID-19 pandemic was declared in March 2020 [2]. At the time of writing this article (July 2021), more than 185 million cases of COVID-19 had been reported across 223 countries that resulted in some 4 million deaths [3]. There is no specific treatment available for COVID-19, and the management of the disease is empirical [4]. To date, only three vaccines have been approved by the US FDA: Pfizer-BioNTech, Moderna and Janssen (Johnson & Johnson) COVID-19 vaccines [5]. However, other vaccines have been approved in other countries [6].

Real-time reverse transcriptase-PCR (RT-PCR) performed on nasopharyngeal or oropharyngeal swabs is the most widely used diagnostic method for SARS-CoV-2 infection [7]. However, this technique has been criticized for being relatively invasive and associated with an increased risk of cross-infection [8]. Serological tests based on the detection of SARS-CoV-2-specific antibodies, IgM and/or IgG may also be used to diagnose COVID-19 [9]. IgM detection can be interpreted as an indicator of acute infection, whereas IgG detection represents previous infection/immunity [9]. However, serological tests have low sensitivity when performed in the early days after the onset of symptoms, have significant rates of false-negative results and show poor result validation [9]. Chest computed tomography is an alternative test for diagnosing and monitoring COVID-19 [10]. Finally, some inflammatory (procalcitonin, C-reactive protein), hematologic (lymphocyte, thrombocytes) and biochemical (creatine kinase-MB, troponin I, D-dimer, aspartate aminotransferase, alanine aminotransferase, lactate dehydrogenase and Γ-glutamyltransferase) biomarkers have been associated with severe COVID-19 and might help in the prognostic risk stratification of patients with COVID-19 [11]. Therefore, the identification of effective diagnostic biomarkers and predictors of COVID-19 severity are increasingly enabling patients to receive accurate and targeted therapy [12].

miRNAs are small (~22-nucleotide long) noncoding RNAs that enhance mRNA degradation and inhibit protein translation [13], play essential regulatory roles in several biological processes and are potential disease biomarkers [13,14]. miRNAs may be useful in diagnosing diseases, evaluating their prognosis, providing potential therapeutic targets and improving our understanding of the physiopathology and signaling pathways involved in diseases [15]. Since viral infection may change host miRNA expression [16] and dysregulated miRNAs have already been studied as biomarkers of several infectious diseases [17], it is expected that miRNAs can also serve as biomarkers of COVID-19 [18–21]. This review aimed to identify which dysregulated miRNAs could serve as biomarkers of COVID-19 and their specific roles.

## Materials & methods

This scoping review was conducted following the recommendations of the Preferred Reporting Items for Systematic reviews and Meta-Analyses statement for Scoping Reviews (PRISMA-ScR) [22]; the review protocol has been registered in the Open Science Framework (https://doi.org/10.17605/OSF.IO/M5VJ6).

### Search strategy

A comprehensive search of literature published before 13 June 2021 was performed using PubMed, PubMed Central, BVS/BIREME, Web of Science, Scopus, EBSCOhost, ProQuest, Embase and Cochrane Library databases to identify relevant studies. The search strategy included a combination of terms related to COVID-19 and miRNAs; the full search strategy can be found in Supplementary Appendix One no language restrictions were imposed.

### Study selection

Cell, animal and human studies that described dysregulated miRNAs in COVID-19 were included. Studies with a purely computational approach and RNA-seq from databases were excluded. Preprints, books and book chapters, editorials, comments, conference proceedings or abstracts and literature reviews and guidelines were also excluded. The studies retrieved from the databases were examined using the Rayyan QCRI program [23] to exclude duplicate files (Phase I), analyze the titles and abstracts of the articles (Phase II) and analyze complete articles of the previously selected abstracts (Phase III). Two reviewers (MBV and ASN) independently reviewed the titles and abstracts of all studies identified by the searches and discussed and addressed any discrepancies arising with a third reviewer (PM). In addition, references cited in all included articles were reviewed to identify any studies that might have been missed.

### Data extraction & analysis

For each included study, details of the author, date of publication or online availability, country, publication type, population, samples, methods used to identify miRNAs, the time when miRNAs were analyzed, miRNAs differentially expressed in SARS-CoV-2-infected cells or animals and patients with COVID-19 (or exposed to SARS-CoV-2), pathophysiological implications of dysregulated miRNAs and main conclusions on the role of miRNAs as biomarkers of COVID-19 were extracted. Two reviewers (MBV and ASN) independently completed data extraction using a preformatted Microsoft Excel spreadsheet. Disagreements were resolved by a third reviewer (PM).

The results of this scoping review are presented using the narrative synthesis approach. Following the PRISMA-ScR guidelines [22], no quality assessment was performed because scoping reviews aim to identify all the available evidence and highlight their main characteristics regardless of the evidence quality.

### Bioinformatics analysis

To generate the interaction network of selected miRNAs, we employed miRTargetLink 2.0, a tool containing experimentally validated interactions on human miRNA–mRNA pairs. Data shown correspond to miRNA-target interactions with strong support, in other words, validated experimentally by reporter assay, western blot, qPCR, microarray and/or next-generation sequencing experiments. The software obtains miRNAs annotations from the latest version of miRBase (v.22.1), while the experimentally validated targets are retrieved from miRTarBase (v.8) and miRATBase. miRTargetLink 2.0 can be freely accessible from the following link (https://ccb-compute.cs.uni-saarland.de/mirtargetlink2/).

## Results

### Search results

**Figure 1.**
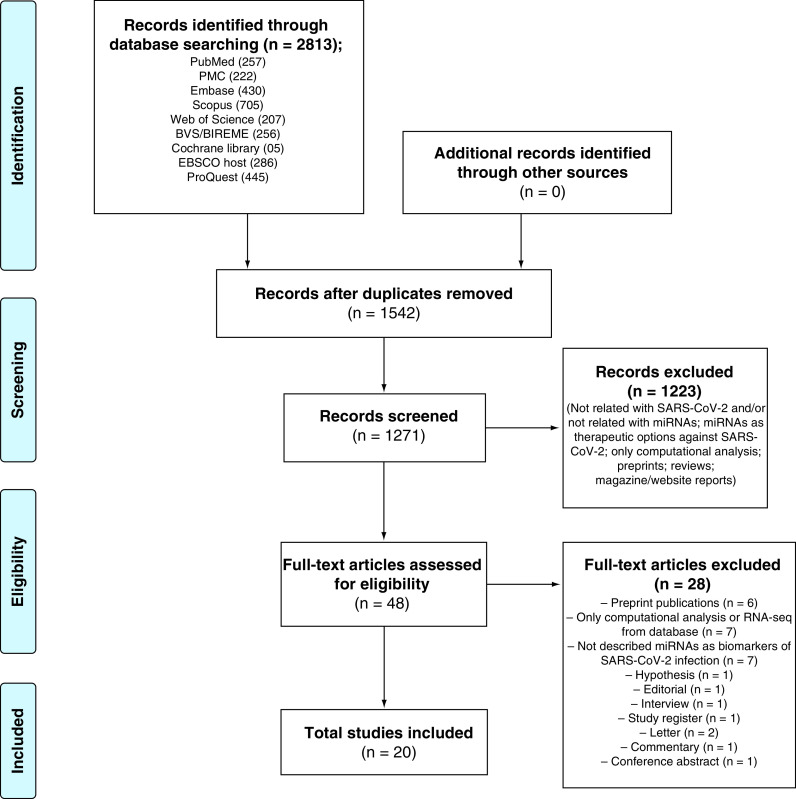
Study selection flowchart through literature search.

An electronic search identified 2813 potentially relevant studies. After removing duplicates and reviewing the titles and abstracts, 48 articles were selected for full-text reading. In addition, no relevant studies were identified by searching the reference lists of the selected studies. After careful full-text reading, 20 studies [24–43] met the inclusion criteria and were thus included in the review. A flowchart of the literature search is shown in Figure 1. The references for the excluded studies, along with the reasons for their exclusion, are available in Supplementary Appendix two.

### Characteristics of the included studies

The characteristics of the 20 studies included in this scoping review are summarized in Table 1. All studies were published in English and reported between October 2020 and June 2021. Moreover, four studies were conducted in cells [24–27], one in an animal model [28] and 15 in humans [29–43]. Seventeen studies were published as research/original articles [24–34,38–42], one as a technical report [43], one as a rapid report [37] and one as correspondence [35]. Nine of these studies were conducted in China (Table 1) [24,29–32,35,38–40].

**Table 1.** Characteristics of the studies included in this scoping review.

Study	Date of publication (or online availability)	Country	Publication type	Population	Ref.
**Cell study**					
Liu *et al.*	Jan 2021	China	Original Article	Vero E6 cell line infected with SARS-CoV-2 and controls (to verify differently expressed virus-encoded miRNAs) and BEAS-b2 cells (to validate the regulatory role of the virus-encoded miRNAs on human genes).	[24]
Wyler *et al.*	Mar 2021	Germany	Original Article	Calu-3 cells infected with SARS-CoV-2 and controls.	[25]
Mishra and Banerjea	Apr 2021	India	Original Research	SARS-CoV-2 Spike gene transfected HEK-293T cells and controls (to quantify miRNAs in released exosomes – ‘exosomes donor cells’) and CHME3 cells (to assess the protein expression levels of miRNAs target genes – ‘exosome recipient microglia’).	[26]
Recchiuti *et al.*	Apr 2021	Italy	Research Article	Macrophages from monocytes culture of peripheral blood obtained from adult volunteers with or without cystic fibrosis. Macrophages exposed to SARS-CoV-2 virion spike 1 glycoprotein (S1) and controls.	[27]
**Animal study**					
Kim *et al.*	Nov 2020	Korea	Research Article	Hamsters infected with SARS-CoV-2 (n = 5) and uninfected control hamsters (n = 5).	[28]
**Human studies**					
Li *et al.*	Oct 2020	China	Research Article	Patients with mild or moderate COVID-19 (n = ten; gender: four male/six female; mean age: 44.9 years) and healthy volunteers (n = four; gender: two male/two female; mean age: 44.8 years).	[29]
Tang *et al.*	Oct 2020	China	Research Article	Patients with moderate (n = six; gender: four male/two female; range age: 20–89 years) and severe (n = six; gender: five male/one female; range age: 60–89 years) COVID-19 and healthy volunteers (n = four; gender: two male/two female; range age: 50–69 years).	[30]
Chen *et al.*	Dec 2020	China	Research Article	Patients with mild (mild or moderate disease (n = 50; gender: 28 male/22 female; mean age: 46.8 years)) and severe (severe or critical disease (n = 16; gender: 12 male/four female; mean age: 65.9 years)) COVID-19 and healthy volunteers (n = 17; gender: nine male/eight female; mean age: 32.9 years).	[31]
Zheng *et al.*	Dec 2020	China	Research Article	Patients with mild (n = six; gender: four male/two female; mean age: 23.4 years) and moderate (n = seven; gender: three male/four female; mean age: 49.1 years) and severe (n = five; gender: four male/one female; mean age: 58.0 years) COVID-19.	[32]
Sabbatinelli *et al.*	Dec 2020	Italy	Research Article	Patients with COVID-19 with multifocal interstitial pneumonia and requiring oxygen therapy (n = 29; gender: 17 male/12 female; these patients were divided in two groups for other objective and the mean age for general group was not shown) and healthy volunteers (n = 29; mean age: 64.1 years).	[33]
Garg *et al.*	Jan 2021	Germany	Research Article	Two cohorts: 1) Discovery cohort: mechanically ventilated COVID-19 patients (n = 18; gender: 17 male/one female; median age: 59 years) and healthy volunteers (n = 15; gender: 14 male/one female; median age: 31 years). 2) Validation cohort: mechanically ventilated COVID-19 patients (n = 20; gender: 14 male/six female; median age: 59.5 years), invasively ventilated influenza-induced ARDS patients (n = 13, gender: 11 male/two female; median age: 56 years) and healthy volunteers (n = 32; gender: 20 male/12 female; median age: 50 years).	[34]
Yang *et al.*	Feb 2021	China	Correspondence	Patients with COVID-19 (n = five) and healthy volunteers (n = three).	[35]
Bagheri-Hosseinabadi *et al.*	Mar 2021	Iran	Original Article	Patients with COVID-19 (n = 33; gender: 13 male/20 female; mean age: 62.4 years) and healthy volunteers (n = 29; gender: nine male/20 female; mean age: 56.6 years).	[36]
Centa *et al.*	Mar 2021	Brazil	Rapid Report	Patients who died due to ARDS, DAD, and multiple organs failure by SARS–CoV-2 infection (n = nine; gender: six male/three female; mean age: 73.4 years) and patients who died due to other causes, not involving lung injuries (n = ten; gender: seven male/three female; mean age: 42.3 years).	[37]
Li *et al.*	Mar 2021	China	Research Paper	Recovered COVID-19 patients (mild/moderate disease (n = 30; gender: 16 male/14 female; median age: 48.0 years) and severe/critical disease (n = 16; gender: 13 male/three female; median age: 54.0 years)) and healthy volunteers (n = 24; gender: ten male/14 female; median age: 36.0 years).	[38]
Mi *et al.*	Mar 2021	China	Research Paper	Fracture patients with IgG (-) (n = 50) and IgG (+) (n = 30) to SARS-CoV-2.	[39]
Li *et al.*	Apr 2021	China	Research Article	Patients with COVID-19 (n = ten; gender: four male/six female; mean age: 44.9 years) and healthy volunteers (n = four; gender one male/three female; mean age: 34.8 years).	[40]
Donyavi *et al.*	Apr 2021	Iran	Research Article	Patients with COVID-19 (n = 18; gender: nine male/nine female; mean age: 38.2 years) and healthy volunteers (n = 15; gender: eight male/seven female; mean age: 36.6 years).	[41]
Gonzalo-Calvo *et al.*	May 2021	Spain	Original Research Article	Two cohorts: 1) Patients with COVID-19 admitted to the pneumology, infectious diseases or internal medicine wards without requiring critical care (n = 43; gender: 18 male/25 female; mean age: 68.0 years) or admitted to the ICU (n = 36; gender: 26 male/ten female; mean age: 68.0 years). 2) Patients with COVID-19 admitted to ICU nonsurvivors (n = 16; gender: 11 male/five female; mean age: 70.5 years) and survivors (n = 20; gender: 15 male/five female; mean age: 60.0 years).	[42]
Mitchell *et al.*	Jun 2021	USA	Technical Report	Patients with mild (n = 13; gender: seven male/six female; mean age: 56.2 years) and severe (n = 17; gender: 15 male/two female; mean age: 69.1 years) COVID-19.	[43]

ARDS: Acute respiratory distress syndrome; BEAS-b2: Human pulmonary epithelial cell; Calu-3: Human epithelial lung cancer cell; CHME3: Human microglial cell; DAD: Diffuse alveolar damage; HEK-293T: Human embryonic kidney cell; ICU: Intensive care unit; n: Number of animals or subjects; Vero E6: African green monkey kidney cell.

Overall, the human studies included a small number of participants. Of the 15 studies, 12 used control groups (healthy volunteers or patients without COVID-19 or not exposed to SARS-CoV-2) for the comparisons [29–31,33–41], while three studies included only patients with COVID-19 [32,42,43]. Interestingly, one study also compared patients with COVID-19 and patients with influenza-induced acute respiratory distress syndrome (ARDS) [34]. Six studies stratified the group of patients with COVID-19 into subgroups by severity [30–32,38,42,43], while one included only patients with mild/moderate disease [29], three included only severely affected and/or critically ill patients [33,34,37] and four did not report the disease severity [35,36,40,41]. One study included patients who underwent orthopedic surgery and who had previously been infected with SARS-CoV-2 (IgG [+]) (Table 1) [39].

### miRNAs as biomarkers of COVID-19

Of the four studies in cells, three quantified differentially expressed miRNAs in cells [24–27] and one in exosomes released from cells [26]. The only animal model study used lung tissue [28]. In most human studies, analysis was performed to identify miRNAs in samples derived from blood. Four used plasma [31,35,36,42], three used serum [33,34,43], one of which also used small extracellular vesicles from whole serum [43], three used whole peripheral blood [29,38,40], two used peripheral blood mononuclear cells [32,41] and one used red blood cells [30], in other words, circulating miRNAs; however, one used lung tissue [37] and the other did not specify the sample used [39] (Supplementary Appendix three). The main results of the studies involving miRNAs as biomarkers of COVID-19 are shown in Supplementary Appendix three.

Regarding the methods used to identify miRNAs, six studies used only sequencing to determine differentially expressed miRNAs [29–32,35,40], while three used quantitative PCR to confirm results previously obtained by sequencing [25,43] or microarray [39] and 11 used PCR to quantify miRNAs previously chosen by bioinformatic analysis or literature search [24,26–28,33,34,36–38,41,42] (Supplementary Appendix three).

*In vitro* studies analyzed miRNAs after treatment with SARS-CoV-2 S1 recombinant protein for 3 h [27], 4 h [25], 12 h [25], 24 h [25] and 48 h [24] after virus infection or 48 h after spike plasmid transfection [26]. The only animal study quantified miRNAs on the 4th day after infection [28]. Of the 15 human studies, three did not report the time when miRNAs were analyzed [30,34,40]. The other 12 reported this information (some were more specific and others less): samples were collected within 1 week after diagnosis [29], at baseline (∼10 days of onset of symptoms) [33], at hospital admission [36], before or following admission to the clinical ward or the intensive care unit [42], at the time of hospitalization [43], over 5 weeks [31], at stages 1–4 of disease progression [35], at the three clinical stages (treatment, convalescence and rehabilitation) [32], during the acute period of the disease and in the recovery period (4–5 weeks after the acute phase) [41], post hospital discharge (in the disease recovery) [38], at the time of hospital admission with a fracture [39] and postmortem [37] (Supplementary Appendix three).

**Figure 2.**
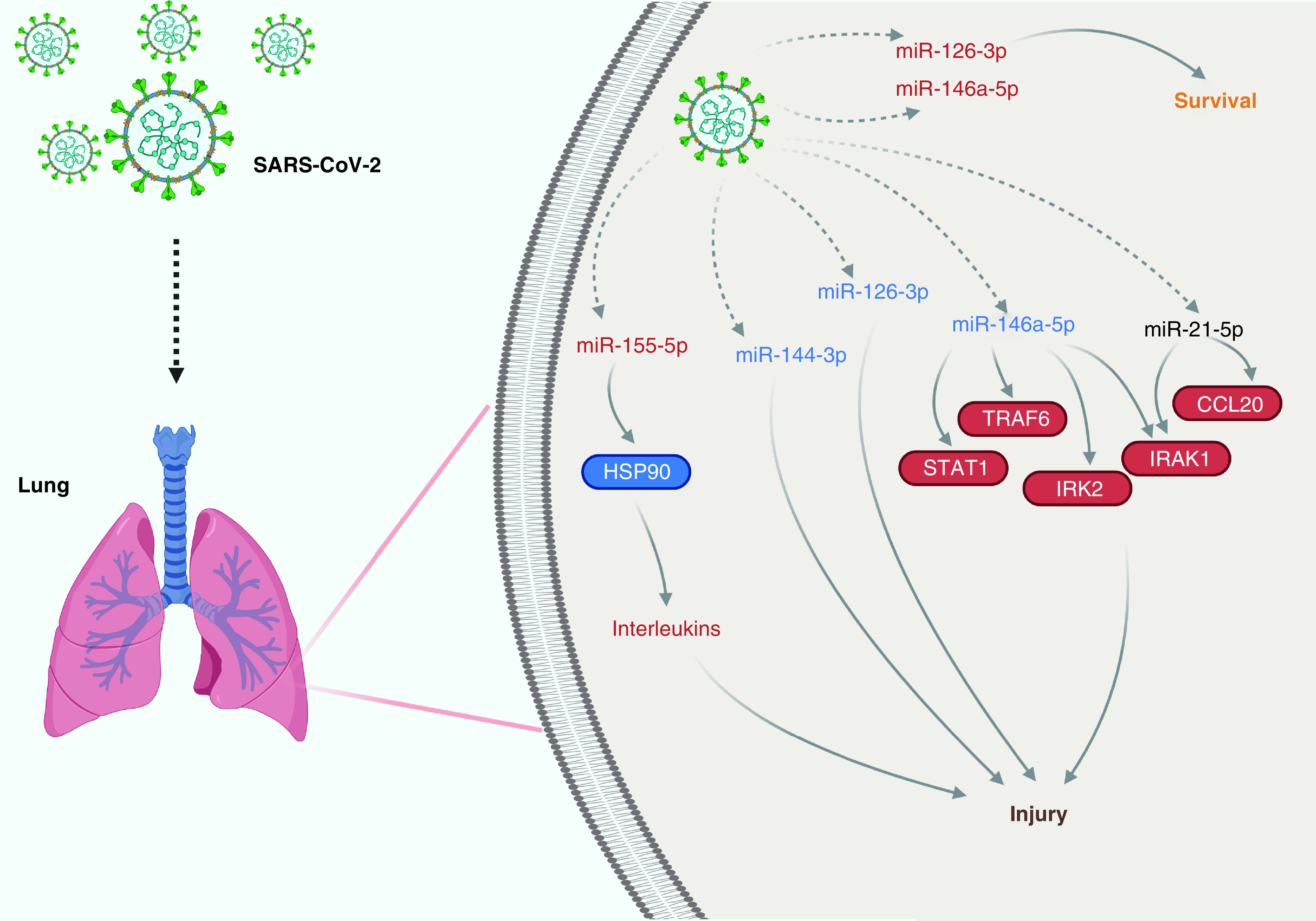
General mechanisms of miRNAs function in COVID-19.

Many miRNAs were significantly dysregulated in COVID-19 (Supplementary Appendices three & four). All studies quantified host miRNAs, except for one that analyzed virus-encoded miRNAs [24]. Host miR-21-5p [30,33,34,40], miR-146a [30,33,43], miR-126-3p [33,34,43], miR-144 [29,35,38] and miR-155 [25,34,38,41] have been shown to be dysregulated in more than two studies (miR-627-5p appears in three studies [29,40,43], but in two of them the patients were the same [29,43], so it was not considered). Among these miRNAs, circulating miR-21-5p (downregulated or upregulated), miR-144 (downregulated) and miR-155 (downregulated or upregulated) appear to be the main potential diagnostic biomarkers and miR-146a (downregulated) appeared to be the biomarker for the severity of COVID-19. In addition to being useful for diagnosis and analysis of severity, miRNAs may also be useful as predictors of mortality, biomarkers of stage/phase and therapeutic targets of COVID-19 (Supplementary Appendix four). Dysregulated miRNAs appear to play key roles in the viral replication and proliferation of infected cells, immune response, inflammation, cardiovascular dysfunction, hyperactivation of human microglia and osteogenic differentiation and bone remodeling, contributing to the pathogenesis of COVID-19 and its sequelae (Supplementary Appendix three). Figure 2 summarizes the general mechanisms of miRNAs function in COVID-19. The main genes regulated by these miRNAs, after bioinformatics analysis, are shown in Supplementary Appendices five–nine.

## Discussion

### Summary of evidence

We aimed to identify miRNAs as biomarkers of COVID-19 through a literature review. The main findings were as follows: the most important miRNAs, identified as potential candidate biomarkers, were miR-21-5p, miR-146a, miR-126-3p, miR-144 and miR-155; circulating miR-21-5p, miR-144 and miR-155 appear to be the main potential diagnostic biomarkers; miR-146a may serve as a biomarker of disease severity; the dysregulated miRNAs appear to play key roles in viral replication and proliferation of infected cells, immune response, inflammation, cardiovascular dysfunction, hyperactivation of human microglia and osteogenic differentiation and bone remodeling; miRNAs may serve as diagnostic biomarkers, indicators of disease severity, predictors of mortality, biomarkers of stage/phase and therapeutic targets of COVID-19. To the best of our knowledge, this is the first scoping review of differentially expressed miRNAs as biomarkers of COVID-19.

### General view of the studies

In our view, the COVID-19 pandemic has changed the profile of published articles. More articles are being published as rapid or short communications containing little information. We also noted that many articles only commented on previously published articles, extrapolating the results for SARS-CoV-2 infection or were attempting to elucidate mechanisms, suggesting possibly important aspects of SARS-CoV-2 infection; we found that many studies used only bioinformatics data. Although, these characteristics are justified by the urgency demanded by the pandemic and the need for knowledge about this new virus, such articles were excluded from our analysis.

Thus, we included only 20 studies in this scoping review despite the emergence of COVID-19 over a year ago, in December 2019 [1]. Most studies included in this scoping review were original/research articles [24–34,36,38–42] and conducted in humans [29–43]. Studies on miRNAs as biomarkers have mostly been conducted in cells and animal models. Since miRNAs as biomarkers can be detected in several tissues and body fluids [15], the human studies included in our review used blood and lung tissue samples [24–38,40–43].

Most of the studies were conducted in China [24,29–32,35,38–40] since this country was the first to be affected by SARS-CoV-2 [1]. We encourage further primary studies to be conducted mainly in North and South American populations where COVID-19 has a high incidence, utilizing a great opportunity for new research and development.

### miRNAs as biomarkers of COVID-19

All the included studies reported dysregulated miRNAs probably induced by COVID-19, indicating that miRNAs are promising diagnostic biomarkers, indicators of disease severity, predictors of mortality, indicators of stage/phase of disease and therapeutic targets; miRNAs may be useful in the diagnosis, prognosis, monitoring and clinical management of COVID-19 and its consequences. Dysregulated miRNAs are related to viral replication, proliferation of infected cells, immune response, inflammation, cardiovascular dysfunction, hyperactivation of human microglia, osteogenic differentiation and bone remodeling [24–43]. Worldwide research has demonstrated that the pathogenesis of COVID-19 is associated with hyper inflammation and intensification of immune effects [44,45]; neurological and musculoskeletal sequelae, among others, can occur in patients with COVID-19 [46].

The most important miRNAs as biomarkers of COVID-19 appear to be miR-21-5p, miR-146a, miR-126-3p, miR-144 and miR-155. Four studies indicated that miR-21-5p expression was dysregulated (downregulated in three studies) in patients with COVID-19 compared with that in healthy volunteers [30,33,34,40] and thus may be a diagnostic biomarker. However, in one study, it was also significantly dysregulated in severe COVID-19 compared with that in moderate COVID-19 [30]. Moreover, this miRNA appeared to be more specific for SARS-CoV-2 infection since it was significantly more dysregulated in patients with COVID-19 than in patients with influenza-induced ARDS [34]. miR-21-5p regulates inflammation and is a marker of a proinflammatory state [33]. In a study that evaluated miRNAs as markers of cardiovascular damage in patients with COVID-19, miR-21-5p was associated with cardiac fibrosis and might be a predictor of chronic myocardial damage and inflammation in COVID-19 survivors [34]. Therefore, miR-21-5p may also be a potential therapeutic target for the management of COVID-19 and the prevention of its cardiovascular consequences.

Three studies found that miR-144 was expressed at lower levels in patients with COVID-19 than in healthy volunteers, revealing a potential biomarker for diagnosis [29,35,40]. However, these three studies used only sequencing technology to identify miRNAs and did not comment on the pathophysiological implications of miR-144.

Four studies revealed that miR-155 expression was dysregulated in COVID-19 [25,34,38,41]. An *in vitro* study showed that the upregulation of miR-155 expression was related to lung injury induced by SARS-CoV-2 and hence may be a potential therapeutic target [25]. Moreover, a human study [34] that compared dysregulated circulating miRNAs between patients with severe COVID-19 and healthy volunteers, as well as patients with severe COVID-19 compared with patients with influenza-induced ARDS, revealed that miR-155 expression was upregulated in both cases; thus, this miRNA may be specific for the diagnosis of SARS-CoV-2-induced ARDS. It might also be a predictor of chronic myocardial damage and inflammation in COVID-19 survivors [34]. Two other human studies indicated that miR-155 plays an important role in the immune response [38,41]. Interestingly, one study found that miR-155 expression was upregulated in patients with mild/moderate COVID-19 compared with that in patients with severe/critical COVID-19 and healthy volunteers, indicating that it is beneficial in controlling SARS-CoV-2 infection (an antiviral response) [38]. Another study indicated that miR-155-5p was a useful marker for discriminating between control and COVID-19 patients by receiver operating characteristic curve analysis [41]. Therefore, miR-155-5p may be used as a potential diagnostic biomarker [41] and may be associated with the progression to severe/critical COVID-19 [38].

Three studies showed that the expression of miR-146a [30,33,43] and miR-126-3p [33,34,43], all biomarkers of a proinflammatory state [33], was significantly downregulated in severe COVID-19. It has been shown that the downregulation of miR-146a expression promotes the inflammatory process since miR-146a-5p is negatively correlated with downstream target mRNA IL-1 receptor-associated kinases 1 and 2 (IRAK1 and IRAK2) and TRAF6 that participate in the NF-κB proinflammatory pathway [30]. In addition, a decline in miR-146a-5p levels may lead to the release of IL-6 [33]. miR-126-3p is a key regulator of endothelial inflammation [47]. Therefore, low levels of miR-146a and miR-126-3p indicate severe COVID-19 and may predict poor outcomes among those who develop systemic hyper inflammation [30,33,34,43].

Apart from those highlighted in this literature review, other miRNAs may also play important roles in COVID-19; these include miR-15b-5p [28,30] and members of the let-7 family (let-7a-5p, let-7b-3p, let-7b-5p, let-7d-5p, let-7f-5p) [27,31,32,41]. The precise roles of these miRNAs and their dysregulation might be clarified in future studies.

### Limitations of the published studies

Cell lines used as *in vitro* models of COVID-19 are essential for the discovery of potential specific therapeutic targets; however, they might respond differently when compared with cells in an organism and; therefore, the findings of this study need to be validated in *in vivo* models [24,25]. Moreover, findings from animal studies need to be validated in humans as much as possible to increase the level of evidence. Regarding human studies; although, it is important to standardize the time of sample collection for analysis after infection or symptom onset, we observed that there was no standard time for the analysis of miRNAs between studies, and some studies did not specify the time elapsed since the onset of symptoms or diagnosis. Further research studies with standardized times need to be conducted, including more prospective longitudinal studies, to verify how these miRNAs behave over the time of infection and disease stage. Moreover, in some human studies, miRNAs described as potential biomarkers were detected by sequencing and need to be validated through RT-PCR in a higher number of patients. Since comorbidities (e.g., obesity, Type 2 diabetes and cardiovascular diseases) can influence the expression of miRNAs in the included studies, it is recommended that comorbidities be precisely matched between the interest and control groups, in addition to age and sex [30]. Finally, to validate the specificity of these miRNAs as biomarkers, future studies should include non-COVID-19 patients with pneumonia or ARDS as positive controls for moderate and severe disease groups as well as asymptomatic COVID-19 patients [30].

### Limitations of this scoping review

Some studies may have been missed because they were not indexed in the searched databases. In addition, this review did not analyze the quality of the studies, considering the inherent characteristics of scoping reviews.

## Conclusion

Based on the 20 included studies, the most important dysregulated miRNAs identified in the selected articles that may play a key role in COVID-19 pathogenesis were miR-21-5p, miR-146a, miR-126-3p, miR-144 and miR-155. Among these miRNAs, miR-21-5p, miR-144 and miR-155 appear to be the main potential diagnostic biomarkers and miR-146a appear to be biomarker of disease severity. In addition, miRNAs may be predictors of mortality, biomarkers of stage/phase and therapeutic targets of COVID-19, as they play key roles in viral replication, proliferation of infected cells, immune response, inflammation, cardiovascular dysfunction, hyperactivation of human microglia, osteogenic differentiation and bone remodeling. Further primary studies that demonstrate the role of miRNAs as biomarkers of SARS-CoV-2 infection/COVID-19 are needed.

## Future perspective

This review provides insights into the role of miRNAs as biomarkers in COVID-19 and the current status and future directions for research in this field. To date, few studies have evaluated miRNAs as biomarkers of COVID-19. However, in the next few years, more studies are expected to be conducted and published, particularly based on populations of the North and South Americas. New miRNA biomarkers in other human biological samples (e.g., saliva) are expected to be discovered. In addition, miRNAs identified by sequencing must be validated by RT-PCR in a larger cohort and more studies including non-COVID-19 patients with pneumonia or ARDS as positive controls for moderate and severe disease groups as well as asymptomatic COVID-19 patients are required. Finally, the findings on miRNAs as biomarkers provide scope for research in the development of effective treatments for COVID-19.

**Summary points**

**Search results**

- A total of 2813 records were identified, 20 of which met the eligibility criteria.

**Characteristics of the included studies**

- All 20 studies were mostly research articles published in English between October 2020 and June 2021 and were mostly conducted in China.
- Fifteen studies were conducted in humans, four using cells and one in an animal model.

**miRNAs as biomarkers of COVID-19**

- miRNAs identified as the most important were miR-21-5p, miR-146a, miR-126-3p, miR-144 and miR-155.
- Among these miRNAs, miR-21-5p, miR-144 and miR-155 appear to be the main diagnostic biomarkers and miR-146a appear to be biomarker of disease severity.
- miRNAs may also be useful as predictors of mortality, biomarkers of stage/phase and therapeutic targets of COVID-19.

**Future directions**

- More studies are needed to investigate and validate the role of miRNAs as biomarkers of COVID-19.
