# Supplementary material for: Role of miRNAs as biomarkers of COVID-19: a scoping review of the status and future directions for research in this field
Source: Biomark Med. 2021 Nov 17:10.2217/bmm-2021-0348. doi: 10.2217/bmm-2021-0348 (PMC8601154; doi:10.2217/bmm-2021-0348)
Supplement: Supplementary file 1 [file Appendix_S1.docx]

Appendix S1

A. PUBMED

#1 ((MicroRNAs[MeSH Terms]) OR (MicroRNAs[Title/Abstract] OR "MicroRNA"[Title/Abstract] OR miRNAs[Title/Abstract] OR "Micro RNA"[Title/Abstract] OR "RNA, Micro"[Title/Abstract] OR miRNA[Title/Abstract] OR "Primary MicroRNA"[Title/Abstract] OR "MicroRNA, Primary"[Title/Abstract] OR "Primary miRNA"[Title/Abstract] OR "miRNA, Primary"[Title/Abstract] OR "pri-miRNA"[Title/Abstract] OR "pri miRNA"[Title/Abstract] OR "RNA, Small Temporal"[Title/Abstract] OR "Temporal RNA, Small"[Title/Abstract] OR stRNA[Title/Abstract] OR "Small Temporal RNA"[Title/Abstract] OR "pre-miRNA"[Title/Abstract] OR "pre miRNA"[Title/Abstract]))

#2 (((("2019 novel coronavirus Pneumonia" OR "2019-novel coronavirus Pneumonia" OR "2019 novel coronavirus Epidemic" OR "2019 novel coronavirus Outbreak" OR "2019 novel coronavirus Pandemic" OR "2019-nCoV Acute Respiratory Disease" OR "2019-nCoV Epidemic" OR "2019-nCoV Outbreak" OR "2019-nCoV Pandemic" OR "2019-nCoV Pneumonia" OR "2019-novel coronavirus (2019-nCoV) Infection" OR "2019­new coronavirus Epidemic" OR "2019­20 China Pneumonia Outbreak" OR "2019­20 Wuhan coronavirus Outbreak" OR "COVID-19" OR "Coronavirus Infection" OR "Infection, Coronavirus" OR "Infections, Coronavirus" OR "MERS (Middle East Respiratory Syndrome) " OR "Middle East Respiratory Syndrome" OR "Novel Coronavirus Pneumonia" OR "Wuhan Seafood Market Pneumonia" OR "Wuhan coronavirus Epidemic" OR "Wuhan coronavirus Infection" OR "Wuhan coronavirus Outbreak" OR "Wuhan coronavirus Pandemic" OR "Wuhan coronavirus Pneumonia") OR ((Coronavirus Infections[MeSH Terms]) OR ("Coronavirus Infections"[Title/Abstract] OR "Coronavirus Infection"[Title/Abstract] OR "Infection, Coronavirus"[Title/Abstract] OR "Infections, Coronavirus"[Title/Abstract] OR "Middle East Respiratory Syndrome"[Title/Abstract] OR "MERS (Middle East Respiratory Syndrome)"[Title/Abstract]))) OR (("COVID-19" [Supplementary Concept]) OR ("COVID-19"[Title/Abstract] OR "2019 novel coronavirus disease"[Title/Abstract] OR "COVID19"[Title/Abstract] OR "COVID-19 pandemic"[Title/Abstract] OR "SARS-CoV-2 infection"[Title/Abstract] OR "COVID-19 virus disease"[Title/Abstract] OR "2019 novel coronavirus infection"[Title/Abstract] OR "2019-nCoV infection"[Title/Abstract] OR "coronavirus disease 2019"[Title/Abstract] OR "coronavirus disease-19"[Title/Abstract] OR "2019-nCoV disease"[Title/Abstract] OR "COVID-19 virus infection"[Title/Abstract]))) OR ("SARS-CoV" OR "SARS-CoV2" OR "2019-nCoV disease" OR "2019-nCoV infection" OR "COVID 19" OR "COVID 2019" OR "nCoV 2019 disease" OR "nCoV 2019 infection" OR "novel coronavirus 2019 disease" OR "novel coronavirus 2019 infection" OR "novel coronavirus disease 2019" OR "novel coronavirus infection 2019" OR "Wuhan coronavirus disease" OR "Wuhan coronavirus infection"))

#1 AND #2

B. PUBMED PMC

#1 ((MicroRNAs[MeSH Terms]) OR (MicroRNAs[Title/Abstract] OR "MicroRNA"[Title/Abstract] OR miRNAs[Title/Abstract] OR "Micro RNA"[Title/Abstract] OR "RNA, Micro"[Title/Abstract] OR miRNA[Title/Abstract] OR "Primary MicroRNA"[Title/Abstract] OR "MicroRNA, Primary"[Title/Abstract] OR "Primary miRNA"[Title/Abstract] OR "miRNA, Primary"[Title/Abstract] OR "pri-miRNA"[Title/Abstract] OR "pri miRNA"[Title/Abstract] OR "RNA, Small Temporal"[Title/Abstract] OR "Temporal RNA, Small"[Title/Abstract] OR stRNA[Title/Abstract] OR "Small Temporal RNA"[Title/Abstract] OR "pre-miRNA"[Title/Abstract] OR "pre miRNA"[Title/Abstract]))

#2 (((("2019 novel coronavirus Pneumonia" OR "2019-novel coronavirus Pneumonia" OR "2019 novel coronavirus Epidemic" OR "2019 novel coronavirus Outbreak" OR "2019 novel coronavirus Pandemic" OR "2019-nCoV Acute Respiratory Disease" OR "2019-nCoV Epidemic" OR "2019-nCoV Outbreak" OR "2019-nCoV Pandemic" OR "2019-nCoV Pneumonia" OR "2019-novel coronavirus (2019-nCoV) Infection" OR "2019­new coronavirus Epidemic" OR "2019­20 China Pneumonia Outbreak" OR "2019­20 Wuhan coronavirus Outbreak" OR "COVID-19" OR "Coronavirus Infection" OR "Infection, Coronavirus" OR "Infections, Coronavirus" OR "MERS (Middle East Respiratory Syndrome) " OR "Middle East Respiratory Syndrome" OR "Novel Coronavirus Pneumonia" OR "Wuhan Seafood Market Pneumonia" OR "Wuhan coronavirus Epidemic" OR "Wuhan coronavirus Infection" OR "Wuhan coronavirus Outbreak" OR "Wuhan coronavirus Pandemic" OR "Wuhan coronavirus Pneumonia") OR ((Coronavirus Infections[MeSH Terms]) OR ("Coronavirus Infections"[Title/Abstract] OR "Coronavirus Infection"[Title/Abstract] OR "Infection Coronavirus"[Title/Abstract] OR "Infections, Coronavirus"[Title/Abstract] OR "Middle East Respiratory Syndrome"[Title/Abstract] OR "MERS (Middle East Respiratory Syndrome)"[Title/Abstract]))) OR (("COVID-19" [Supplementary Concept]) OR ("COVID-19"[Title/Abstract] OR "2019 novel coronavirus disease"[Title/Abstract] OR "COVID19"[Title/Abstract] OR "COVID-19 pandemic"[Title/Abstract] OR "SARS-CoV-2 infection"[Title/Abstract] OR "COVID-19 virus disease"[Title/Abstract] OR "2019 novel coronavirus infection"[Title/Abstract] OR "2019-nCoV infection"[Title/Abstract] OR "coronavirus disease 2019"[Title/Abstract] OR "coronavirus disease-19"[Title/Abstract] OR "2019-nCoV disease"[Title/Abstract] OR "COVID-19 virus infection"[Title/Abstract]))) OR ("SARS-CoV" OR "SARS-CoV2" OR "2019-nCoV disease" OR "2019-nCoV infection" OR "COVID 19" OR "COVID 2019" OR "nCoV 2019 disease" OR "nCoV 2019 infection" OR "novel coronavirus 2019 disease" OR "novel coronavirus 2019 infection" OR "novel coronavirus disease 2019" OR "novel coronavirus infection 2019" OR "Wuhan coronavirus disease" OR "Wuhan coronavirus infection"))

#1 AND #2

C. BVS/BIREME

#1 (micrornas OR "MicroRNA" OR mirnas OR "Micro RNA" OR "RNA, Micro" OR mirna OR "Primary MicroRNA" OR "MicroRNA, Primary" OR "Primary miRNA" OR "miRNA, Primary" OR "pri-miRNA" OR "pri miRNA" OR "RNA, Small Temporal" OR "Temporal RNA, Small" OR strna OR "Small Temporal RNA" OR "pre-miRNA" OR "pre miRNA")

2# ("2019 novel coronavirus Pneumonia" OR "2019-novel coronavirus Pneumonia" OR "2019 novel coronavirus Epidemic" OR "2019 novel coronavirus Outbreak" OR "2019 novel coronavirus Pandemic" OR "2019-nCoV Acute Respiratory Disease" OR "2019-nCoV Epidemic" OR "2019-nCoV Outbreak" OR "2019-nCoV Pandemic" OR "2019-nCoV Pneumonia" OR "2019-novel coronavirus (2019-nCoV) Infection" OR "2019­new coronavirus Epidemic" OR "2019­20 China Pneumonia Outbreak" OR "2019­20 Wuhan coronavirus Outbreak" OR "COVID-19" OR "Coronavirus Infection" OR "Infection, Coronavirus" OR "Infections, Coronavirus" OR "MERS (Middle East Respiratory Syndrome) " OR "Middle East Respiratory Syndrome" OR "Novel Coronavirus Pneumonia" OR "Wuhan Seafood Market Pneumonia" OR "Wuhan coronavirus Epidemic" OR "Wuhan coronavirus Infection" OR "Wuhan coronavirus Outbreak" OR "Wuhan coronavirus Pandemic" OR "Wuhan coronavirus Pneumonia" OR "Coronavirus Infections" OR "Coronavirus Infection" OR "Infection, Coronavirus" OR "Infections, Coronavirus" OR "Middle East Respiratory Syndrome" OR "MERS (Middle East Respiratory Syndrome)" OR "COVID-19" OR "2019 novel coronavirus disease" OR "COVID19" OR "COVID-19 pandemic" OR "SARS-CoV-2 infection" OR "COVID-19 virus disease" OR "2019 novel coronavirus infection" OR "2019-nCoV infection" OR "coronavirus disease 2019" OR "coronavirus disease-19" OR "2019-nCoV disease" OR "COVID-19 virus infection" OR "SARS-CoV" OR "SARS-CoV2" OR "2019-nCoV disease" OR "2019-nCoV infection" OR "COVID 19" OR "COVID 2019" OR "nCoV 2019 disease" OR "nCoV 2019 infection" OR "novel coronavirus 2019 disease" OR "novel coronavirus 2019 infection" OR "novel coronavirus disease 2019" OR "novel coronavirus infection 2019" OR "Wuhan coronavirus disease" OR "Wuhan coronavirus infection")

#1 AND #2

D. Web of Science

#1 TÓPICO: (MicroRNAs OR "MicroRNA" OR miRNAs OR "Micro RNA" OR "RNA, Micro" OR miRNA OR "Primary MicroRNA" OR "MicroRNA, Primary" OR "Primary miRNA" OR "miRNA, Primary" OR "pri-miRNA" OR "pri miRNA" OR "RNA, Small Temporal" OR "Temporal RNA, Small" OR stRNA OR "Small Temporal RNA" OR "pre-miRNA" OR "pre miRNA") Índices=SCI-EXPANDED, SSCI, A&HCI, CPCI-S, CPCI-SSH, ESCI Tempo estipulado=Todos os anos

#2 Todos os campos: ("2019 novel coronavirus Pneumonia" OR "2019-novel coronavirus Pneumonia" OR "2019 novel coronavirus Epidemic" OR "2019 novel coronavirus Outbreak" OR "2019 novel coronavirus Pandemic" OR "2019-nCoV Acute Respiratory Disease" OR "2019-nCoV Epidemic" OR "2019-nCoV Outbreak" OR "2019-nCoV Pandemic" OR "2019-nCoV Pneumonia" OR "2019-novel coronavirus (2019-nCoV) Infection" OR "2019­new coronavirus Epidemic" OR "2019­20 China Pneumonia Outbreak" OR "2019­20 Wuhan coronavirus Outbreak" OR "COVID-19" OR "Coronavirus Infection" OR "Infection, Coronavirus" OR "Infections, Coronavirus" OR "MERS (Middle East Respiratory Syndrome) " OR "Middle East Respiratory Syndrome" OR "Novel Coronavirus Pneumonia" OR "Wuhan Seafood Market Pneumonia" OR "Wuhan coronavirus Epidemic" OR "Wuhan coronavirus Infection" OR "Wuhan coronavirus Outbreak" OR "Wuhan coronavirus Pandemic" OR "Wuhan coronavirus Pneumonia") OR TÓPICO: "Coronavirus Infections" OR "Coronavirus Infection" OR "Infection, Coronavirus" OR "Infections, Coronavirus" OR "Middle East Respiratory Syndrome" OR "MERS (Middle East Respiratory Syndrome) ") OR TÓPICO: ("COVID-19" OR "2019 novel coronavirus disease" OR "COVID19" OR "COVID-19 pandemic" OR "SARS-CoV-2 infection" OR "COVID-19 virus disease" OR "2019 novel coronavirus infection" OR "2019-nCoV infection" OR "coronavirus disease 2019" OR "coronavirus disease-19" OR "2019-nCoV disease" OR "COVID-19 virus infection") OR Todos os campos: ("SARS-CoV" OR "SARS-CoV2" OR "2019-nCoV disease" OR "2019-nCoV infection" OR "COVID 19" OR "COVID 2019" OR "nCoV 2019 disease" OR "nCoV 2019 infection" OR "novel coronavirus 2019 disease" OR "novel coronavirus 2019 infection" OR "novel coronavirus disease 2019" OR "novel coronavirus infection 2019" OR "Wuhan coronavirus disease" OR "Wuhan coronavirus infection") Índices=SCI-EXPANDED, SSCI, A&HCI, CPCI-S, PCI-SSH, ESCI Tempo estipulado=Todos os anos

#1 AND #2

E. SCOPUS

#1 ( TITLE-ABS-KEY ( micrornas OR "MicroRNA" OR mirnas OR "Micro RNA" OR "RNA, Micro" OR mirna OR "Primary MicroRNA" OR "MicroRNA, Primary" OR "Primary miRNA" OR "miRNA, Primary" OR "pri-miRNA" OR "pri miRNA" OR "RNA, Small Temporal" OR "Temporal RNA, Small" OR strna OR "Small Temporal RNA" OR "pre-miRNA" OR "pre miRNA" ) )

#2 ( ALL ( "2019 novel coronavirus Pneumonia" OR "2019-novel coronavirus Pneumonia" OR "2019 novel coronavirus Epidemic" OR "2019 novel coronavirus Outbreak" OR "2019 novel coronavirus Pandemic" OR "2019-nCoV Acute Respiratory Disease" OR "2019-nCoV Epidemic" OR "2019-nCoV Outbreak" OR "2019-nCoV Pandemic" OR "2019-nCoV Pneumonia" OR "2019-novel coronavirus (2019-nCoV) Infection" OR "2019­new coronavirus Epidemic" OR "2019­20 China Pneumonia Outbreak" OR "2019­20 Wuhan coronavirus Outbreak" OR "COVID-19" OR "Coronavirus Infection" OR "Infection, Coronavirus" OR "Infections, Coronavirus" OR "MERS (Middle East Respiratory Syndrome) " OR "Middle East Respiratory Syndrome" OR "Novel Coronavirus Pneumonia" OR "Wuhan Seafood Market Pneumonia" OR "Wuhan coronavirus Epidemic" OR "Wuhan coronavirus Infection" OR "Wuhan coronavirus Outbreak" OR "Wuhan coronavirus Pandemic" OR "Wuhan coronavirus Pneumonia" ) OR TITLE-ABS-KEY ( "Coronavirus Infections" OR "Coronavirus Infection" OR "Infection, Coronavirus" OR "Infections, Coronavirus" OR "Middle East Respiratory Syndrome" OR "MERS (Middle East Respiratory Syndrome)" ) OR TITLE-ABS-KEY ( "COVID-19" OR "2019 novel coronavirus disease" OR "COVID19" OR "COVID-19 pandemic" OR "SARS-CoV-2 infection" OR "COVID-19 virus disease" OR "2019 novel coronavirus infection" OR "2019-nCoV infection" OR "coronavirus disease 2019" OR "coronavirus disease-19" OR "2019-nCoV disease" OR "COVID-19 virus infection" ) OR ALL ( "SARS-CoV" OR "SARS-CoV2" OR "2019-nCoV disease" OR "2019-nCoV infection" OR "COVID 19" OR "COVID 2019" OR "nCoV 2019 disease" OR "nCoV 2019 infection" OR "novel coronavirus 2019 disease" OR "novel coronavirus 2019 infection" OR "novel coronavirus disease 2019" OR "novel coronavirus infection 2019" OR "Wuhan coronavirus disease" OR "Wuhan coronavirus infection" ) )

#1 AND #2

F. EBSCOHOST

#1 ( MicroRNAs OR "MicroRNA" OR miRNAs OR "Micro RNA" OR "RNA, Micro" OR miRNA OR "Primary MicroRNA" OR "MicroRNA, Primary" OR "Primary miRNA" OR "miRNA, Primary" OR "pri-miRNA" OR "pri miRNA" OR "RNA, Small Temporal" OR "Temporal RNA, Small" OR stRNA OR "Small Temporal RNA" OR "pre-miRNA" OR "pre miRNA" )

#2 ( "2019 novel coronavirus Pneumonia" OR "2019-novel coronavirus Pneumonia" OR "2019 novel coronavirus Epidemic" OR "2019 novel coronavirus Outbreak" OR "2019 novel coronavirus Pandemic" OR "2019-nCoV Acute Respiratory Disease" OR "2019-nCoV Epidemic" OR "2019-nCoV Outbreak" OR "2019-nCoV Pandemic" OR "2019-nCoV Pneumonia" OR "2019-novel coronavirus (2019-nCoV) Infection" OR "2019­new coronavirus Epidemic" OR "2019­20 China Pneumonia Outbreak" OR "2019­20 Wuhan coronavirus Outbreak" OR "COVID-19" OR "Coronavirus Infection" OR "Infection, Coronavirus" OR "Infections, Coronavirus" OR "MERS (Middle East Respiratory Syndrome) " OR "Middle East Respiratory Syndrome" OR "Novel Coronavirus Pneumonia" OR "Wuhan Seafood Market Pneumonia" OR "Wuhan coronavirus Epidemic" OR "Wuhan coronavirus Infection" OR "Wuhan coronavirus Outbreak" OR "Wuhan coronavirus Pandemic" OR "Wuhan coronavirus Pneumonia" OR "Coronavirus Infections" OR "Coronavirus Infection" OR "Infection, Coronavirus" OR "Infections, Coronavirus" OR "Middle East Respiratory Syndrome" OR "MERS (Middle East Respiratory Syndrome)" OR "COVID-19" OR "2019 novel coronavirus disease" OR "COVID19" OR "COVID-19 pandemic" OR "SARS-CoV-2 infection" OR "COVID-19 virus disease" OR "2019 novel coronavirus infection" OR "2019-nCoV infection" OR "coronavirus disease 2019" OR "coronavirus disease-19" OR "2019-nCoV disease" OR "COVID-19 virus infection" OR "SARS-CoV" OR "SARS-CoV2" OR "2019-nCoV disease" OR "2019-nCoV infection" OR "COVID 19" OR "COVID 2019" OR "nCoV 2019 disease" OR "nCoV 2019 infection" OR "novel coronavirus 2019 disease" OR "novel coronavirus 2019 infection" OR "novel coronavirus disease 2019" OR "novel coronavirus infection 2019" OR "Wuhan coronavirus disease" OR "Wuhan coronavirus infection" )

#1 AND #2

G. PROQUEST

#1 (MicroRNAs OR "MicroRNA" OR miRNAs OR "Micro RNA" OR "RNA, Micro" OR miRNA OR "Primary MicroRNA" OR "MicroRNA, Primary" OR "Primary miRNA" OR "miRNA, Primary" OR "pri-miRNA" OR "pri miRNA" OR "RNA, Small Temporal" OR "Temporal RNA, Small" OR stRNA OR "Small Temporal RNA" OR "pre-miRNA" OR "pre miRNA")

#2 (("2019 novel coronavirus Pneumonia" OR "2019-novel coronavirus Pneumonia" OR "2019 novel coronavirus Epidemic" OR "2019 novel coronavirus Outbreak" OR "2019 novel coronavirus Pandemic" OR "2019-nCoV Acute Respiratory Disease" OR "2019-nCoV Epidemic" OR "2019-nCoV Outbreak" OR "2019-nCoV Pandemic" OR "2019-nCoV Pneumonia" OR "2019-novel coronavirus (2019-nCoV) Infection" OR "2019­new coronavirus Epidemic" OR "2019­20 China Pneumonia Outbreak" OR "2019­20 Wuhan coronavirus Outbreak" OR "COVID-19" OR "Coronavirus Infection" OR "Infection, Coronavirus" OR "Infections, Coronavirus" OR "MERS (Middle East Respiratory Syndrome) " OR "Middle East Respiratory Syndrome" OR "Novel Coronavirus Pneumonia" OR "Wuhan Seafood Market Pneumonia" OR "Wuhan coronavirus Epidemic" OR "Wuhan coronavirus Infection" OR "Wuhan coronavirus Outbreak" OR "Wuhan coronavirus Pandemic" OR "Wuhan coronavirus Pneumonia") OR ("Coronavirus Infections" OR "Coronavirus Infection" OR "Infection, Coronavirus" OR "Infections, Coronavirus" OR "Middle East Respiratory Syndrome" OR "MERS (Middle East Respiratory Syndrome)") OR ("COVID-19" OR "2019 novel coronavirus disease" OR "COVID19" OR "COVID-19 pandemic" OR "SARS-CoV-2 infection" OR "COVID-19 virus disease" OR "2019 novel coronavirus infection" OR "2019-nCoV infection" OR "coronavirus disease 2019" OR "coronavirus disease-19" OR "2019-nCoV disease" OR "COVID-19 virus infection") OR ("SARS-CoV" OR "SARS-CoV2" OR "2019-nCoV disease" OR "2019-nCoV infection" OR "COVID 19" OR "COVID 2019" OR "nCoV 2019 disease" OR "nCoV 2019 infection" OR "novel coronavirus 2019 disease" OR "novel coronavirus 2019 infection" OR "novel coronavirus disease 2019" OR "novel coronavirus infection 2019" OR "Wuhan coronavirus disease" OR "Wuhan coronavirus infection"))

#1 AND #2

H. EMBASE

#1 ('microrna'/exp OR 'microrna'/syn)

#2 ('coronavirus infection'/syn OR '2019 novel coronavirus pneumonia' OR '2019-novel coronavirus pneumonia' OR '2019 novel coronavirus epidemic' OR '2019 novel coronavirus outbreak' OR '2019 novel coronavirus pandemic' OR '2019-ncov acute respiratory disease' OR '2019-ncov epidemic' OR '2019-ncov outbreak' OR '2019-ncov pandemic' OR '2019-ncov pneumonia' OR '2019-novel coronavirus (2019-ncov) infection' OR '2019­new coronavirus epidemic' OR '2019­20 china pneumonia outbreak' OR '2019­20 wuhan coronavirus outbreak' OR 'coronavirus infection'/exp OR 'coronavirus infection' OR 'infection, coronavirus' OR 'infections, coronavirus' OR 'mers (middle east respiratory syndrome)' OR 'middle east respiratory syndrome'/exp OR 'middle east respiratory syndrome' OR 'novel coronavirus pneumonia' OR 'wuhan seafood market pneumonia' OR 'wuhan coronavirus epidemic' OR 'wuhan coronavirus outbreak' OR 'wuhan coronavirus pandemic' OR 'wuhan coronavirus pneumonia' OR 'covid-19'/exp OR 'covid-19' OR '2019 novel coronavirus disease' OR 'covid19'/exp OR 'covid19' OR 'covid-19 pandemic' OR 'sars-cov-2 infection' OR 'covid-19 virus disease' OR '2019 novel coronavirus infection' OR 'coronavirus disease-19' OR 'covid-19 virus infection' OR 'coronavirus disease 2019'/exp OR 'coronavirus disease 2019' OR 'coronavirus disease 2019'/syn OR 'sars-cov'/exp OR 'sars-cov' OR 'sars-cov2' OR '2019-ncov disease'/exp OR '2019-ncov disease' OR '2019-ncov infection'/exp OR '2019-ncov infection' OR 'covid 19'/exp OR 'covid 19' OR 'covid 2019'/exp OR 'covid 2019' OR 'ncov 2019 disease'/exp OR 'ncov 2019 disease' OR 'ncov 2019 infection'/exp OR 'ncov 2019 infection' OR 'novel coronavirus 2019 disease'/exp OR 'novel coronavirus 2019 disease' OR 'novel coronavirus 2019 infection'/exp OR 'novel coronavirus 2019 infection' OR 'novel coronavirus disease 2019'/exp OR 'novel coronavirus disease 2019' OR 'novel coronavirus infection 2019'/exp OR 'novel coronavirus infection 2019' OR 'wuhan coronavirus disease'/exp OR 'wuhan coronavirus disease' OR 'wuhan coronavirus infection'/exp OR 'wuhan coronavirus infection')

#1 AND #2

I.COCHRANE

#1 **MeSH descriptor: [Coronavirus Infections] explode all trees OR**  **("Coronavirus Infections" OR "Coronavirus Infection" OR "Infection, Coronavirus" OR "Infections, Coronavirus" OR "Middle East Respiratory Syndrome" OR "MERS (Middle East Respiratory Syndrome)"):ti,ab,kw OR**  **("COVID-19" OR "2019 novel coronavirus disease" OR "COVID19" OR "COVID-19 pandemic" OR "SARS-CoV-2 infection" OR "COVID-19 virus disease" OR "2019 novel coronavirus infection" OR "2019-nCoV infection" OR "coronavirus disease 2019" OR "coronavirus disease-19" OR "2019-nCoV disease" OR "COVID-19 virus infection"):ti,ab,kw OR ("SARS-CoV" OR "SARS-CoV2" OR "2019-nCoV disease" OR "2019-nCoV infection" OR "COVID 19" OR "COVID 2019" OR "nCoV 2019 disease" OR "nCoV 2019 infection" OR "novel coronavirus 2019 disease" OR "novel coronavirus 2019 infection" OR "novel coronavirus disease 2019" OR "novel coronavirus infection 2019" OR "Wuhan coronavirus disease" OR "Wuhan coronavirus infection") OR**  **("2019 novel coronavirus Pneumonia" OR "2019-novel coronavirus Pneumonia" OR "2019 novel coronavirus Epidemic" OR "2019 novel coronavirus Outbreak" OR "2019 novel coronavirus Pandemic" OR "2019-nCoV Acute Respiratory Disease" OR "2019-nCoV Epidemic" OR "2019-nCoV Outbreak" OR "2019-nCoV Pandemic" OR "2019-nCoV Pneumonia" OR "2019-novel coronavirus (2019-nCoV) Infection" OR "2019­new coronavirus Epidemic" OR "2019­20 China Pneumonia Outbreak" OR "2019­20 Wuhan coronavirus Outbreak" OR "COVID-19" OR "Coronavirus Infection" OR "Infection, Coronavirus" OR "Infections, Coronavirus" OR "MERS (Middle East Respiratory Syndrome) " OR "Middle East Respiratory Syndrome" OR "Novel Coronavirus Pneumonia" OR "Wuhan Seafood Market Pneumonia" OR "Wuhan coronavirus Epidemic" OR "Wuhan coronavirus Infection" OR "Wuhan coronavirus Outbreak" OR "Wuhan coronavirus Pandemic" OR "Wuhan coronavirus Pneumonia")**

**#2 MeSH descriptor: [MicroRNAs] explode all trees OR**  **(MicroRNAs OR "MicroRNA" OR miRNAs OR "Micro RNA" OR "RNA, Micro" OR miRNA OR "Primary MicroRNA" OR "MicroRNA, Primary" OR "Primary miRNA" OR "miRNA, Primary" OR "pri-miRNA" OR "pri miRNA" OR "RNA, Small Temporal" OR "Temporal RNA, Small" OR stRNA OR "Small Temporal RNA" OR "pre-miRNA" OR "pre miRNA"):ti,ab,kw**

#1 AND #2
