# Supplementary material for: Role of miRNAs as biomarkers of COVID-19: a scoping review of the status and future directions for research in this field
Source: Biomark Med. 2021 Nov 17:10.2217/bmm-2021-0348. doi: 10.2217/bmm-2021-0348 (PMC8601154; doi:10.2217/bmm-2021-0348)
Supplement: Supplementary file 2 [file R1_Appendix_2.docx]

Appendix S2

| Reason for exclusion | Authors, year | Title | Reference |
| --- | --- | --- | --- |
| Preprints | Ahmed et al., 2020 | Interplay of host regulatory network on SARS-CoV-2 binding and replication machinery | [1] |
|  | Aiewsakun et al., 2020 | SARS-CoV-2 genetic variations associated with COVID-19 severity | [2] |
|  | Alsamman and Zayed, 2020 | The transcriptomic profiling of COVID-19 compared to SARS, MERS, Ebola, and H1N1 * | [3] |
|  | Chan et al., 2020 | Conserved genomic terminals of SARS-COV-2 as co-evolving functional elements and potential therapeutic targets * | [4] |
|  | Islam and Khan, 2020 | SARS-CoV-2 mutations altering regulatory properties: deciphering host’s and virus’s perspectives * | [5] |
|  | Sabbatinelli et al., 2020 | Decreased serum levels of inflammaging marker miR-146a are associated with clinical response to tocilizumab in COVID-19 patients ** | [6] |
| Only computational analysis and RNA-seq from databases | Ahmed et al., 2020 | Regulatory cross talk between SARS-COV-2 receptor binding and replication machinery in the human host | [7] |
|  | Arora et al., 2020 | Unravelling host-pathogen interactions: ceRNA network in SARS-CoV-2 infection (COVID-19) | [8] |
|  | Bertolazzi et al., 2020 | Mir-1207-5p can contribute to dysregulation of inflammatory response in Covid-19 via targeting SARS-COV-2 RNA | [9] |
|  | Chow and Salmena, 2020 | Prediction and analysis of SARS-CoV-2-targeting microRNA in human lung epithelium | [10] |
|  | Pierce et al., 2020 | Computational analysis of targeting SARS-COV-2, viral entry proteins ACE2 and TMPRSS2, and interferon genes by host microRNAs | [11] |
|  | Zarubin et al., 2020 | Structural variability, expression profile, and pharmacogenetic properties of TMPRSS2 gene as a potential target for Covid-19 therapy | [12] |
|  | Mohammadi-Dehcheshmeh et al., 2021 | A transcription regulatory sequence in the 5´untranslated  region of SARS-COV-2 is vital for virus replication with an  altered evolutionary pattern against human inhibitory microRNAs | [13] |
| Not described miRNAs as biomarkers for SARS-CoV-2 infection | Lu et al., 2020 | MicroRNAs targeting the SARS-CoV-2 entry receptor ACE2 in cardiomyocytes | [14] |
|  | Matarese et al., 2020 | Mir-98 regulates TMPRSS2 expression in human endothelial cells: key implications for Covid-19 | [15] |
|  | Sacconi et al., 2020 | TMPRSS2, a SARS-CoV-2 internalization protease is downregulated in head and neck cancer patients | [16] |
|  | Sm and McLellan, 2020 | Implications of SARS-COV-2 mutations for genomic RNA structure and host microRNA targeting | [17] |
|  | Zhang et al., 2021 | Association analysis framework of genetic and exposure risks for COVID-19 in middle-aged and elderly adults | [18] |
|  | He et al., 2021 | MicroRNA-574-5p attenuates acute respiratory distress syndrome by targeting HMGB1 | [19] |
|  | Lee et al., 2021 | Honeysuckle aqueous extracts induced let-7a suppress EV71 replication and pathogenesis in vitro and in vivo and is predicted to inhibit SARS-COV-2 | [20] |
| Hypothesis | Adeoye and Thomson, 2020 | ‘The double-edged sword’ – An hypothesis for Covid-19-induced salivary biomarkers | [21] |
| Editorial | Pontecorvi et al., 2020 | MicroRNAs as new possible actors in gender disparities of Covid-19 pandemic | [22] |
| Interview | NR | Otago researchers discover potential target points on SARS-CoV-2 genome | [23] |
| Study Register | NR | Randomised clinical trial of interventions for the treatment of COVID-19 in the community setting for high risk older people | [24] |
| Letter | Soheilifar, Neghab, and Basiri, 2020 | Biological impacts of microRNAs in Covid-19: implications for anti-viral miRNA-based therapies | [25] |
|  | Xie et al., 2021 | Therapeutic potential of C1632 by inhibition of SARS-CoV-2  replication and viral-induced inflammation through upregulating let-7 | [26] |
| Commentary | Soltani and Zandi, 2021 | MiR‑200c‑3p upregulation and ACE2 downregulation via bacterial LPS and LTA as nteresting aspects for COVID‑19 treatment and immunity | [27] |
| Conference Abstract | Gambardella et al., 2021 | MicroRNAs drive endothelial dysfunction and tromboembolism in COVID-19 | [28] |

Abbreviation: NR – not reported. *Although these preprints had already been published in scientific journals, they were excluded from our scoping review (due to other exclusion criteria). **This preprint has already been published and has been included in our scoping review.

REFERENCES: [1] Ahmed, S. S. S. J.; Paramasivam, P.; Raj, K.; Kumar, V. et al. Interplay of host regulatory network on SARS-CoV-2 binding and replication machinery. **bioRxiv**, p. 2020.2004.2020.050138, 2020. [2] Aiewsakun, P.; Wongtrakoongate, P.; Thawornwattana, Y.; Hongeng, S. et al. SARS-CoV-2 genetic variations associated with COVID-19 severity. **medRxiv**, p. 2020.2005.2027.20114546, 2020. [3] Alsamman, A. M.; Zayed, H. The transcriptomic profiling of COVID-19 compared to SARS, MERS, Ebola, and H1N1. **bioRxiv**, p. 2020.2005.2006.080960, 2020. [4] Chan, A. P.; ChoI, Y.; Schork, N. J. Conserved genomic terminals of SARS-CoV-2 as co-evolving functional elements and potential therapeutic targets. **bioRxiv**, p. 2020.2007.2006.190207, 2020. [5] Islam, A. M. M. K; and Khan, M. A-A-K. SARS-CoV-2 mutations altering regulatory properties: deciphering host's and virus's perspectives. **bioRxiv**, p. 2020.2006.2015.150482, 2020. [6] Sabbatinelli, J.; Giuliani, A.; Matacchione, G.; Latini, S. et al. Decreased serum levels of the inflammaging marker miR-146a are associated with clinical response to tocilizumab in COVID-19 patients. **medRxiv**, p. 2020.2007.2011.20151365, 2020. [7] Ahmed, S. S. S. J.; Paramasivam, P.; Raj, K.; Kumar, V. et al. Regulatory Cross Talk Between SARS-CoV-2 Receptor Binding and Replication Machinery in the Human Host. **Front Physiol**, 11, p. 802, 2020. [8] Arora, S.; Singh, P.; Dohare, R.; Jha, R. et al. Unravelling host-pathogen interactions: ceRNA network in SARS-CoV-2 infection (COVID-19). **Gene**, 762, p. 145057, Dec 2020. [9] Bertolazzi, G.; Cipollina, C.; Benos, P. V.; Tumminello, M. et al. miR-1207-5p can contribute to dysregulation of inflammatory response in COVID-19. **Front Cell Infect Microbiol**, 10, p. 586592, 2020. [10] Chow, J. T.; Salmena, L. Prediction and analysis of SARS-COV-2-targeting microRNA in human lung epithelium. **Genes (Basel)**, 11, n. 9, 08 2020. [11] Pierce, J. B.; Simion, V.; Icli, B.; Pérez-Cremades, D. et al. Computational analysis of targeting SARS-CoV-2, viral entry proteins ACE2 and TMPRSS2, and interferon genes by host microRNAs. **Genes (Basel)**, 11, n. 11, 11 2020. [12] Zarubin A, Stepanov V, Markov A, Kolesnikov N, Marusin A, Khitrinskaya I, Swarovskaya M, Litvinov S, Ekomasova N, Dzhaubermezov M, Maksimova N, Sukhomyasova A, Shtygasheva O, Khusnutdinova E, Radzhabov M, Kharkov V. Structural variability, expression profile, and pharmacogenetic properties of TMPRSS2 Gene as a potential target for COVID-19 therapy. **Genes (Basel)**, 2020 Dec 25;12(1):19. doi: 10.3390/genes12010019. PMID: 33375616; PMCID: PMC7823984. [13] Mohammadi-Dehcheshmeh M, Moghbeli SM, Rahimirad S, Alanazi IO, Shehri ZSA, Ebrahimie E. A Transcription regulatory sequence in the 5' untranslated region of SARS-CoV-2 is vital for virus replication with an altered evolutionary pattern against human inhibitory microRNAs. **Cells**. 2021 Feb 4;10(2):319. doi: 10.3390/cells10020319. PMID: 33557205; PMCID: PMC7913991. [14] Lu, D.; Chatterjee, S.; Xiao, K.; Riedel, I. et al. MicroRNAs targeting the SARS-CoV-2 entry receptor ACE2 in cardiomyocytes. **J Mol Cell Cardiol**, 148, p. 46-49, 11 2020. [15] Matarese, A.; Gambardella, J.; Sardu, C.; Santulli, G. miR-98 regulates TMPRSS2 expression in human endothelial cells: key implications for COVID-19. **Biomedicines**, 8, n. 11, Oct 2020. [16] Sacconi, A.; Donzelli, S.; Pulito, C.; Ferrero, S. et al. TMPRSS2, a SARS-CoV-2 internalization protease is downregulated in head and neck cancer patients. **J Exp Clin Cancer Res**, 39, n. 1, p. 200, Sep 2020. [17] Hosseini Rad SM, A.; Mclellan, A. D. Implications of SARS-CoV-2 mutations for genomic RNA structure and host microRNA targeting. **Int J Mol Sci**, 21, n. 13, Jul 2020. [18] Zhang Y, Yang H, Li S, Li WD, Wang J, Wang Y. Association analysis framework of genetic and exposure risks for COVID-19 in middle-aged and elderly adults. **Mech Ageing Dev**. 2021 Jan 12;194:111433. doi: 10.1016/j.mad.2021.111433. Epub ahead of print. PMID: 33444631; PMCID: PMC7801182.[19] He B, Zhou W, Rui Y, Liu L, Chen B, Su X. MicroRNA-574-5p attenuates acute respiratory distress syndrome by targeting HMGB1. **Am J Respir Cell Mol Biol**. 2021 Feb;64(2):196-207. doi: 10.1165/rcmb.2020-0112OC. PMID: 33202146; PMCID: PMC7874400 [20] Lee YR, Chang CM, Yeh YC, Huang CF, Lin FM, Huang JT, Hsieh CC, Wang JR, Liu HS. Honeysuckle aqueous extracts induced *let-7a* suppress EV71 replication and pathogenesis in vitro and in vivo and is predicted to inhibit SARS-CoV-2. **Viruses**. 2021 Feb 16;13(2):308. doi: 10.3390/v13020308. PMID: 33669264; PMCID: PMC7920029. [21] Adeoye, J.; Thomson, P. 'The double-edged sword' - an hypothesis for Covid-19-induced salivary biomarkers. **Med Hypotheses**, 143, p. 110124, Oct 2020 [22] Pontecorvi, G.; Bellenghi, M.; Ortona, E.; Carè, A. MicroRNAs as new possible actors in gender disparities of Covid-19 pandemic. **Acta Physiol (Oxf)**, 230, n. 1, p. e13538, 09 2020. [23] <https://www.expresshealthcare.in/covid19-updates/otago-researchers-discover-potential-target-points-on-sars-cov-2-genome-mirna/423245/> Accessed in: 12/21/2020 [24] <https://www.anzctr.org.au/Trial/Registration/TrialReview.aspx?id=379801&isReview=true> Accessed in: 12/21/2020. [25] Soheilifar, M. H; Neghab, H. K; AND Basiri, P. Biological impacts of microRNAS in Covid-19: implications for anti-viral miRNA-based therapies. **Arch Clin Infect Dis**, Oct 2020. [26] Xie C, Chen Y, Luo D, Zhuang Z, Jin H, Zhou H, Li X, Lin H, Zheng X, Zhang J, Wang P, Zhao J, Zhao Y, Huang H. Therapeutic potential of C1632 by inhibition of SARS-CoV-2 replication and viral-induced inflammation through upregulating let-7. **Signal Transduct Target Ther**. 2021 Feb 22;6(1):84. doi: 10.1038/s41392-021-00497-4. PMID: 33619243; PMCID: PMC7897876.[27] Soltani S, Zandi M. miR-200c-3p upregulation and ACE2 downregulation via bacterial LPS and LTA as nteresting aspects for COVID-19 treatment and immunity. **Mol Biol Rep**. 2021 May 3:1–2. doi: 10.1007/s11033-021-06378-x. Epub ahead of print. PMID: 33939073; PMCID: PMC8091633. [28] Gambardella J; Morelli M; Sardu C; Maggi P; Matarese A; Marfella R; Messina V; Jankauskas S; Paolisso G; Gaetano Santulli G; MicroRNAs drive endothelial dysfunction and tromboembolism in COVID-19. **European Journal of Heart Failure** (2020) 23 (Supp. S1) 3–13. doi:10.1002/ejhf.2100.
