# Supplementary material for: Role of miRNAs as biomarkers of COVID-19: a scoping review of the status and future directions for research in this field
Source: Biomark Med. 2021 Nov 17:10.2217/bmm-2021-0348. doi: 10.2217/bmm-2021-0348 (PMC8601154; doi:10.2217/bmm-2021-0348)
Supplement: Supplementary file 3 [file R1_Appendix_3.docx]

Appendix S3

| **Author(s)** | **Sample used to identify miRNAs** | **Methods used to identify miRNAs** | **Time when the miRNAs were analyzed** | **Differentially expressed miRNAs in SARS-CoV-2 infected cells/animals or patients with COVID-19 or exposed to the SARS-CoV-2** | **Pathophysiological implications of deregulated miRNAs*** | **Main conclusion on the role of miRNAs as biomarkers of SARS-CoV-2 infection/COVID-19** |
| --- | --- | --- | --- | --- | --- | --- |
| *Cell study* |  |  |  |  |  |  |
| Liu et al. [24] | Cells | qRT-PCR | 48 h after virus infection | Virus encoded miRNAs  ***Upregulated***  miR-147-3p  miR-66-3p  miR-369-3p  miR-359-5p | miR-147-3p and miR-359-5p were predicted to be able to repress the expression of genes involved apoptosis (such as *RAD9A and FOXO3,* respectively), thus reducing the host cell apoptosis to subvert host defense.  In human cells, the overexpression of miR-147-3p resulted in significantly decreased transcript levels of *EXOC7*, *RAD9A*, and *TFE3*. In addition, this miRNA also elevated the average expression of *TMPRSS2* (important for SARS-CoV-2 entry into the cell), but the variation between different replicates was large.  The article did not explain the implications of the other deregulated miRNAs on SARS-CoV-2 infection/COVID-19. | With further experiments to validate the role of candidate miRNAs *in vivo*, there is a reasonable prospect to develop antiviral therapeutics against SARS-CoV-2 through targeting the candidate miRNAs. |
| Wyler et al. [25] | Cells | NGS and  qRT-PCR | 4, 12, and 24 h after virus infection | NGS  ***Downregulated***  miR-940  ***Upregulated***  miR-155-3p  miR-4485  qRT-PCR  ***Upregulated***  miR-155-3p  miR-155-5p  miR-4485 | Lung injury by ARDS was attenuated by deletion of miR-155.  The article did not explain the implications of the other deregulated miRNAs on SARS-CoV-2 infection/COVID-19. | miR-155 may be a potential therapeutic target in the context of COVID-19. |
| Mishra and Banerjea [26] | Exosomes released from cells | qRT-PCR | 48 h after Spike plasmid transfection | ***Upregulated***  miR-590-3p  miR-148a | In human microglia treated with exosomes loaded with miRNAs, target genes *IRF9* and *USP33* were suppressed and, consequently, IRF9 and USP33 protein expression decreased. In addition, the downregulation of USP33 *via* miR-148a was also followed by reduced levels of IRF9 in human microglia. This confirmed the positive regulatory function of USP33 upon IRF9 protein levels. Ultimately, decreased levels of IRF9 was related to upregulation of the pro-inflammatory gene expression (*TNFa, NF-kB and IFN-b*). It leads to central nervous system damage through hyperactivation of human microglia. In summary, SARS-CoV-2 Spike targets the USP33-IRF9 axis *via* exosomal miR-148a to activate human microglia. | This study provides insights regarding the impact of Spike gene on shuttling of host miRNAs via exosomes to trigger the neuroinflammation and have thrown some light on new immune regulatory checkpoints in human microglia which need to be explored further for finding new treatment modalities to combat SARS-CoV-2 neuropathogenesis. |
| Recchiuti et al. [27] | Macrophages | qRT-PCR | After the treatment with SARS-CoV-2 S1 recombinant protein for 3 h | Treated macrophage in relation not treated.  ***Downregulated (macrophages from volunteers with and without cystic fibrosis)***  miR-29a  miR-16  miR-103  miR-125a  ***Upregulated (only in macrophages from volunteers with cystic fibrosis)***  miR-197  let-7b  ***Downregulated (only in macrophages from volunteers with cystic fibrosis)***  miR-21a  miR-223  ***Downregulated (only in macrophages from volunteers without cystic fibrosis)***  let-7b | The miRNAs are associated with inflammation, by regulating the NF-κB. | SARS-CoV-2 triggers common and distinct responses in macrophages from volunteers with cystic fibrosis and from healthy individuals compared a not treated macrophage with a SARS-CoV-2 S1 recombinant protein. |
| *Animal study* |  |  |  |  |  |  |
| Kim et al. [28] | Lung tissues | qRT-PCR | 4 days post-infection. | ***Downregulated***  miR-15b-5p  miR-140-3p  miR-422a  ***Upregulated***  miR-195-5p  miR-221-3p | Downregulation of miR-15b-5p may enable SARS-CoV-2 to escape the host immune defense by inhibiting apoptosis and promote the proliferation of infected cells.  Upregulation of miR-195-5p promotes apoptosis by inducing cell cycle arrest and prevents excessive proliferation of the infected cells as the host immune response.  Upregulation of miR-221-3p can suppress the innate immune system and facilitate virus replication.  Downregulation of miR-140-3p may have supported virus infection by inhibiting apoptosis and promoting cell proliferation.  Downregulation of miR-422a promotes the apoptosis of infected cells by inducing the host immune response. | miR-15b-5p and miR-195-5p, the most differentially expressed compared to other miRNAs, may potentially be diagnostic biomarkers for SARS-CoV-2 infection. |
| *Human studies* |  |  |  |  |  |  |
| Li et al. [29] | Whole peripheral blood | NGS | A single sample was collected for each patient; however, this was done at different times within one week after diagnosis. | 35 miRNAs (upregulated)  38 miRNAs (downregulated)  MiRNAs showing expression FC > 1.3:    ***Downregulated***  miR-183-5p  miR-627-5p  miR-144-3p  ***Upregulated***  miR-16-2-3p  miR-6501-5p  miR-618 | Upregulation of miR-618 is related to dysregulation of immune function.  The article did not explain the implications of the other deregulated miRNAs on SARS-CoV-2 infection/COVID-19. | miR-618 may be a promising therapeutic and diagnostic target to treat COVID-19 patients. |
| Tang et al. [30] | Red blood cell-depleted whole blood | NGS | NR | 1. Severe cases in relation to healthy volunteers (FC ≥ 2):   ***Downregulated***  miR-146a-5p  miR-21-5p  miR-142-3p  miR-181a-2-3p  miR-31-5p  miR-99a-5p  ***Upregulated***  miR-3605-3p  miR-15b-5p  miR-486-5p  miR-486-3p   1. Moderate cases in relation to healthy volunteers (FC ≥ 2):   ***Downregulated***  miR-146a-5p  miR-21-5p  miR-142-3p  ***Upregulated***  miR-3605-3p   1. Severe cases in relation to moderate cases (FC ≥ 2):   ***Downregulated***  miR-146a-5p  miR-21-5p  miR-142-3p  miR-181a-2-3p  miR-31-5p  miR-99a-5p  ***Upregulated***  miR-3605-3p  miR-15b-5p  miR-486-5p  miR-486-3p | These miRNAs are correlated with inflammation and antiviral immune responses:  Downregulation of miR-146a-5p, miR-21-5p, and miR-142-3p promotes the inflammatory process.  Upregulation of miR-15b-5p accelerates intracellular viral replication, mediates virus-induced transcriptome changes, and intensifies the severity of COVID-19.  Upregulation of miR-486-5p and miR-486-3p may result in immune response dysregulation.  Downregulation of miR-181a-2-3p is associated with enhanced TLR4 and CXCL8 expression.  Downregulation of miR-99a-5p is associated with the expression of the proinflammatory genes *IGF1R* and *MTMR3*, inducing weaker antiviral immunity.  The article did not explain the implications of the other deregulated miRNAs on SARS-CoV-2 infection/COVID-19.  . | miR-146a-5p, miR-21-5p, and miR-142-3p are potential biomarkers of COVID-19 severity.  miR-146a-5p, miR-21-5p, and miR-142-3p are novel potential therapeutic targets for COVID-19.  Several miRNAs, such as miR-15b-5p, are specific for severe COVID-19 and may serve as potential biomarkers and therapeutic targets. |
| Chen et al. [31] | Plasma | NGS | Samples were collected over a 5-week period, with 65 patients having more than one sampling timepoint. | Among 769 exRNAs, compared with healthy controls, mild and severely ill patients had significantly different expression patterns (higher or lower) in 39.9% and 20.5% exRNAs, respectively.  The most important dysregulated miRNAs were:  miR-98-5p  let-7a-5p  let-7d-5p  miR-378a-3p  miR-340-5p  let-7f-5p  miR-335-3p | Altered expression of let-7 family members, among exRNAs, revealed the importance of T-cell activation and inflammatory response suppression.  The article did not explain the implications of the other deregulated miRNAs on SARS-CoV-2 infection/COVID-19. | Let-7 family miRNAs (let-7a-5p, let-7d-5p, and let-7f-5p) and  miR-378a-3p, miR-340-5p, miR-335-3p, and miR-98-5p may also be effective predictive biomarkers of clinical outcome at the onset of disease. |
| Zheng et al. [32] | PBMC | NGS | At the three clinical  stages (treatment, convalescence, and rehabilitation). | In total, 67 clinical stage-related differentially expressed miRNAs were identified.  miRNAs significantly deregulated in the rehabilitation stage  ***Downregulated***  let-7b-5p  miR-103a-2-5p  miR-200c-3p  miR-2115-3p | Downregulation of let-7b-5p is associated with upregulation of its target gene *RASGRP1*.  Downregulation of miR-103a-2-5p is associated with upregulation of its target gene *CDK6*.  Downregulation of miR-200c-3p is associated with upregulation of its target gene *ZEB1*.  Downregulation of miR-2115-3p is associated with upregulation of its target gene *ATG5*.  Thus, all these miRNAs regulate T cell differentiation during recovery from COVID-19. | The results showed a robust T-cell immune response, but a weakening innate and humoral immunity during recovery from illness, regardless of the clinical types or disease severity, as  demonstrated by the altered levels of miRNAs let-7b-5p, miR-103a-2-5p, miR-200c-3p, and miR-2115-3p involved in T cell activation and differentiation. |
| Sabbatinelli et al. [33] | Serum | RT-PCR and ddPCR | At baseline (~10 days of onset of symptoms) | ***Downregulated***  miR-146a-5p  miR-21-5p  miR-126-3p | Downregulation of miR-146a-5p may unleash the release of IL-6. The unbalance of the IL-6/miR-146a-5p axis could depend, at least in part, from IL-6-stimulating nuclear factors other than NF-κB or synergistically acting with NF-κB, thus dramatically exacerbating IL-6 synthesis without a concomitant induction of  miR-146a-5p transcription. Patients with COVID-19 showed increased IL-6 levels and reduced miR-146a-5p levels compared to healthy subjects, pointing to an imbalance in the IL-6/miR-146a-5p physiological axis in the pathogenesis of SARS-CoV-2 infection.  The article did not explain the implications of the other deregulated miRNAs on SARS-CoV-2 infection/COVID-19. | miR-146a-5p, miR-21-5p, and miR-126 showed to be biomarkers of a pro-inflammatory state in patients with COVID-19.  Low levels of circulating miR-146a-5p in patients with COVID-19 may predict poor outcome among those who develop systemic hyperinflammation. |
| Garg et al. [34] | Serum | qRT-PCR | NR | 1. Discovery cohort   Patients with COVID-19 in relation to healthy volunteers:  ***Downregulated***  miR-126-3p  ***Upregulated***  miR-21-5p  miR-155-5p  miR-208a-3p  miR-499-5p     1. Validation cohort 2. Patients with COVID-19 in relation to healthy volunteers:   ***Upregulated***  miR-21-5p  miR-155-5p  miR-208a-3p  miR-499-5p   1. Patients with COVID-19 in relation to patients with influenza-induced ARDS patients:   ***Upregulated***  miR-21-5p  miR-155-5p  miR-499-5p | Upregulation of miR-208a and miR-499 were associated with elevated procalcitonin and lactate.  Upregulation of these miRNAs in COVID-19 might be associated inflammation process.  The article did not explain the implications of the other deregulated miRNAs on SARS-CoV-2 infection/COVID-19. | The upregulation of miR-21, miR-155,  miR-208a, and miR-499 in COVID-19 survivors might be predictors of chronic myocardial damage and inflammation.  miRNA profiles were able to differentiate between severely ill Patients with COVID-19 and patients with influenza-induced ARDS, indicating a rather specific response and cardiac involvement of COVID-19. |
| Yang et al.[35] | Plasma | NGS | Stages of disease progression (stages 1 to 4) | ***Downregulated (Top 10)***  miR-19b-1  miR-96  miR-19b-2  miR-451a  miR-451b  miR-194-1  miR-144  miR-486-2  miR-15a  miR-29c  ***Upregulated (Top 10)***  miR-3609  miR-1244-1  miR-663a  miR-3916  miR-3687-2  miR-7846  miR-5047  miR-3184  miR-1248  miR-6891 | In COVID-19 patients, decreased expression of miR-451a/miR-374a may promote expression of IL-6R/CCL2 at the protein level. These results suggest that decreased miR-451a/miR-374a levels may exacerbate IL-6-induced cytokine storms by promoting IL-6R/CCL2 translation in COVID-19 patients.  The article did not explain the implications of the other deregulated miRNAs on SARS-CoV-2 infection/COVID-19. | This study offers insight into the potential mechanism to understand the elevated cytokine storms caused by IL-6 in COVID-19 patients and may shed light on drug development for this new disease. |
| Bagheri-Hosseinabadi et al. [36] | Plasma | qRT-PCR | At hospital admission  (time after the diagnosis do not specified) | ***Downregulated***  miR-10b | Downregulated miR-10b was correlated with increased cytokine levels. As a consequence, miR-10b might be involved in the modulation of inflammation in the COVID-19 cases.  Higher decrease in the miR-10b levels per aging in the COVID-19 patients may be associated with higher inflammation, leading to higher mortality rate and severity of the disease. | miR-10b may contribute to cytokine storm and may have an involvement in the immunopathogenesis of COVID-19. |
| Centa et al. [37] | Lung biopsies | qRT-PCR | Post-mortem | ***Downregulated***  miR-26a-5p  miR-29b-3p  miR-34a-5p  ROC curve analysis: miR-26a-5p and miR-29b-3p were among the miRNAs that showed the best power to discriminate the COVID-19 group from the controls | These three miRNAs were demonstrated by pathway enrichment analysis to be involved in cell-adhesion, adherens junctions, and extracellular matrix receptor interaction signaling pathways, i.e., endothelial function, as well as, in pathways with gene targets that regulate viral diseases. In addition, miR-26a-5p and miR-29b-3p were correlated with tissue expression of some cytokines indicating that their dysregulation may contribute to the inflammatory process, cytokine storm, and endothelial dysfunction. | miR-26a-5p, miR-29b-3p, and miR-34a-5p are involved in endothelial dysfunction and inflammatory response in patients with SARS-CoV-2 infection and the occurrence of severe lung injury and immunothrombosis. |
| Li et al. [38] | Whole peripheral blood | PCR array | Post-hospital discharge (recovery)  (exact time not specified) | In both mild/moderate disease in relation to healthy volunteers analysis and mild/moderate disease in relation to severe/critical disease analysis:  ***Upregulated***  miR-155  miR-130a | SARS-CoV-2-mediated regulation of interferon-stimulated genes may occur partially through miRNAs such as miR-155 and miR-130a and the activation of interferon-stimulated genes appears to be beneficial in controlling SARS-CoV-2 (antiviral response). | Downregulation of miR-155 and miR-130a may be associated with progression to severe/critical COVID-19. A more detailed understanding of the antiviral response is required in order to better predict disease severity and to optimize patient management. |
| Mi et al. [39] | NR | Microarray and  qRT-PCR | Data collected at  the time of hospital admission with fracture  (time after SARS-CoV-2 exposition not specified) | IgG (+) patients compared to the IgG (-) patients   1. Microarray   50 miRNAs deregulated  ***Upregulated (FC > 5.0)***  miR-4485-3p  miR-1973   1. qRT-PCR (validation)   ***Upregulated***  miR-4485-3p | miR-4485-5p negatively regulates bone remodeling *in vitro* and *in vivo*.  TLR4 was identified as a potential downstream regulator of miR-4485-3p.  TLR4 reduction induced by the overexpression of miR-4485 was shown to inhibit osteogenic differentiation and bone remodeling.  The article did not explain the implications of miR-1973 on SARS-CoV-2 infection/COVID-19. | miR-4485-5p is highly enriched in IgG (+) patients, and, as it is involved in fracture repair, can impact the entire healing cascade. Furthermore, TLR signaling was identified as an important potential therapeutic target for improving fracture healing in the SARS-CoV-2-infected population. |
| Li et al. [40] | Whole peripheral blood | NGS | NR | (FC ≥ 2)  ***Downregulated***  miR-183-5p  miR-32-5p  miR-29b-2-5p  miR-3613-5p  miR-18b-5p  miR-96-5p  miR-199b-3p  miR-199a-3p  miR-144-3p  miR-4521  miR-18a-5p  miR-20a-5p  miR-21-5p  miR-627-5p    ***Upregulated***  miR-16-2-3p  miR-5695  miR-10399-3p  miR-6501-5p  miR-4659a-3p  miR-142-5p  miR-505-5p  miR-125b-5p  miR-618 | Functional analysis on the targets of differentially expressed miRNA was involved in the regulation of immune effector process, inflammatory mediator regulation of TRP channels, lung cell differentiation, negative regulation of NIK/NF-kB signaling, and negative regulation of T cell differentiation, which all play significant roles in depressing  viral infection.  Potential pathogenesis of excessive cytokine production such as STRG.119845.30/miR-20a-5p/TNFRSF1B, MSTRG.119845.30/miR-29b-2-5p/FCGR2A, and MSTRG.106112.2/miR-6501-5p/STAT3 axis, which may also play an important role in the development of ground-glass opacity in COVID-19 patients. | This study gives new insights into inflammation regulatory mechanisms of coding and noncoding RNAs in COVID-19, which may provide novel diagnostic biomarkers and therapeutic avenues for COVID-19 patients. |
| Donyavi et al. [41] | PBMC | qRT-PCR | After diagnosis of COVID-19 in the acute period of the disease and in the recovery period (4-5 weeks after the acute phase) | 1. Acute and post-acute patients in relations to control group:   ***Upregulated***  miR-29a-3p  miR-146a-3p  miR-155-5p  let-7b-3p   1. Post-acute patients in relations to acute patients:   ***Upregulated***  miR-29a-3p  miR-146a-3p  let-7b-3p  ROC curve analysis: miR-29a-3p, miR-146a-3p, and miR-155-5p were the useful markers for discriminating between control and acute COVID-19 patients. In addition, miR-29a-3p and miR-146a3p presented good value in distinguishing acute COVID-19 patients from post-acute COVID-9 patients. | The measured miRNAs could be associated with some genes (e.g., NF-kB) that are involved in COVID-19 progression.  The expression of miR-155 is increased due to an immune response to fight with SARS-CoV-2. | miR-29a-3p, miR-155-5p and miR- 146a-3p may be used as a potential diagnostic marker for diagnosis of acute-COVID-19 disease. Moreover, miR-29a-3p and miR-146a-3p can probably act as novel biomarkers for distinguishing post-acute from acute phase of COVID-19 disease and, therefore, for monitoring of COVID-19 disease. |
| Gonzalo-Calvo et al. [42] | Plasma | qRT-PCR | Before or following admission to the clinical ward or the ICU | 1. ICU patients in relation to wards patients   Ten of 41 miRNAs analyzed were differentially detected:  ***Downregulated***  miR-16-5p  miR-92a-3p  miR-150-5p  miR-451a  miR-486-5p  ***Upregulated***  miR-27a-3p  miR-27b-3p  miR-148a-3p  miR-199a-5p  miR-491-5p  ROC curve analysis: a 3-miRNA panel was able to discriminate the severity with high accuracy (miR-148a-3p, miR-486-5p and miR-451a).   1. Patients admitted to ICU nonsurvivors in relation to survivors   Six of 41 miRNAs analyzed were differentially detected:  ***Downregulated***  miR-16-5p,  miR-92a-3p  miR-98-5p  miR-132-3p  miR-192-5p  miR-323a-3p  ROC curve analysis: a 2-miRNA panel was able to discriminate between survivors and nonsurvivors with high accuracy (miR-192-5p and miR-323a-3p). | The article did not explain the implications of deregulated miRNAs on SARS-CoV-2 infection/COVID-19. | Severe COVID-19 induces characteristic molecular changes in the circulating miRNA profile. MiRNAs, particularly a signature composed of miR-192-5p and miR-323a-3p, are relevant predictors of patient outcome in the clinically severe phase. |
| Mitchell et al. [43] | Serum  Small-EV from whole serum | NGS and qRT-PCR | Serum collection at time of hospitalization  (time after the diagnosis not specified) | 1. Severe cases in relation to mild cases 2. Small-EV   NGS  ***Downregulated***  miR-146a  miR-126-3p  miR-151-3p  miR-126-5p  ***Upregulated***  miR-15a  miR-424  miR-627-5p  miR-145  miR-205  miR-200c  qRT-PCR (validation of Top 4)  ***Downregulated***  miR-146a  miR-126-3p   1. Serum   NGS  ***Upregulated***  miR-550-5p  miR-629 | The observations that both miR-146a and miR-126-3p may be downregulated in severely ill COVID-19 hospitalized patients on mechanical ventilators, when compared to mildly ill hospitalized patients, are consistent with reported anti-inflammatory and vascular health-promoting properties of these regulators.  The article did not explain the implications of the other deregulated miRNAs on SARS-CoV-2 infection/COVID-19. | Downregulated expression of miR-146a and miR-126-3p in small-EV may be associated with severity of the COVID-19. |

Abbreviations: ARDS, acute respiratory distress syndrome; COVID-19, coronavirus disease 2019; ddPCR, droplet digital polymerase chain reaction; exRNAs, extracellular RNAs; FC, fold change; ICU, intensive care unit; IgG, immunoglobulin G; miR, microRNA; miRNAs, microRNAs; NGS, next-generation sequencing; NR, not reported; PBMC, peripheral blood mononuclear cells; qRT-PCR, quantitative real-time polymerase chain reaction; ROC, receiver operating characteristic; SARS-CoV-2, severe acute respiratory syndrome coronavirus 2; Small-EV, small-extracellular vesicle; TLR, toll-like receptor. *Quotes from other authors were not considered.
