# Supplementary figures and images for: Role of miRNAs as biomarkers of COVID-19: a scoping review of the status and future directions for research in this field
Source: Biomark Med. 2021 Nov 17:10.2217/bmm-2021-0348. doi: 10.2217/bmm-2021-0348 (PMC8601154; doi:10.2217/bmm-2021-0348)

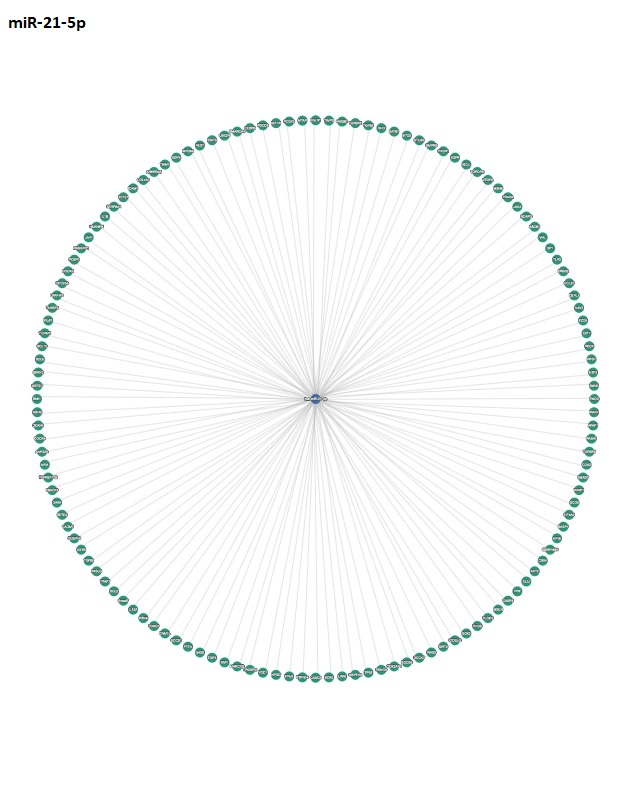

Supplement: Supplementary file 5 [file R1_Appendix_5.jpg]

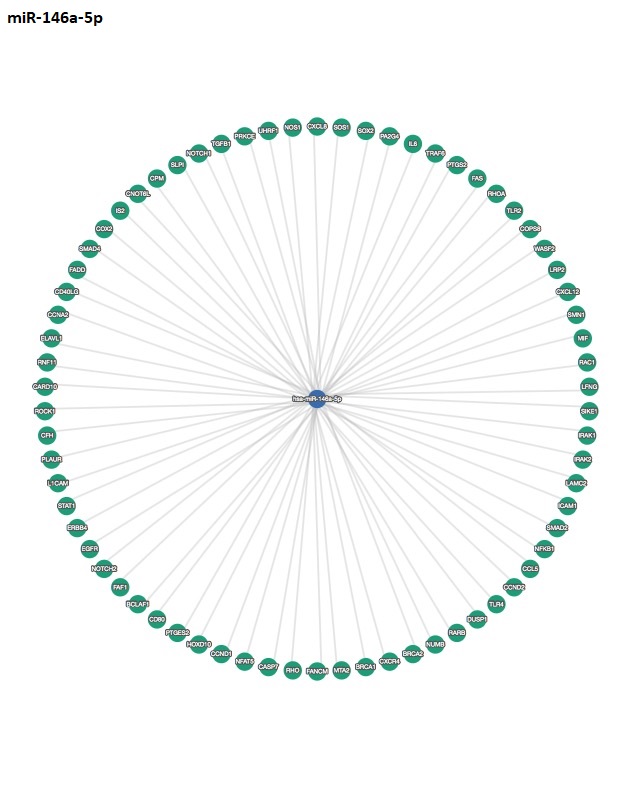

Supplement: Supplementary file 6 [file R1_Appendix_6.jpg]

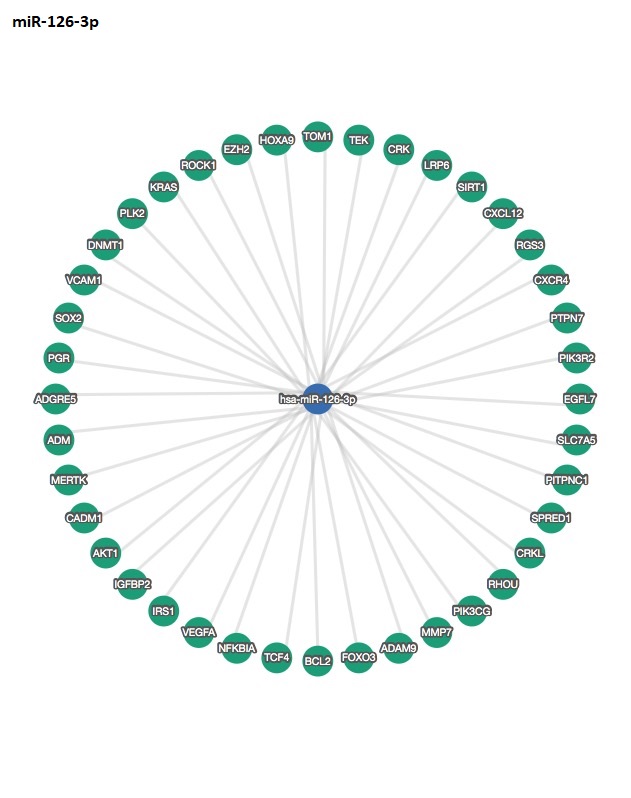

Supplement: Supplementary file 7 [file R1_Appendix_7.jpg]

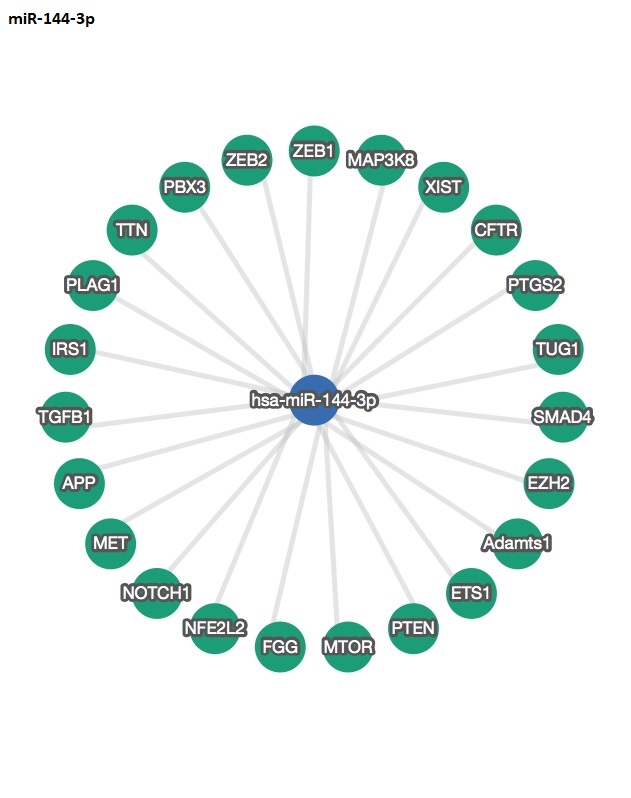

Supplement: Supplementary file 8 [file R1_Appendix_8.jpg]

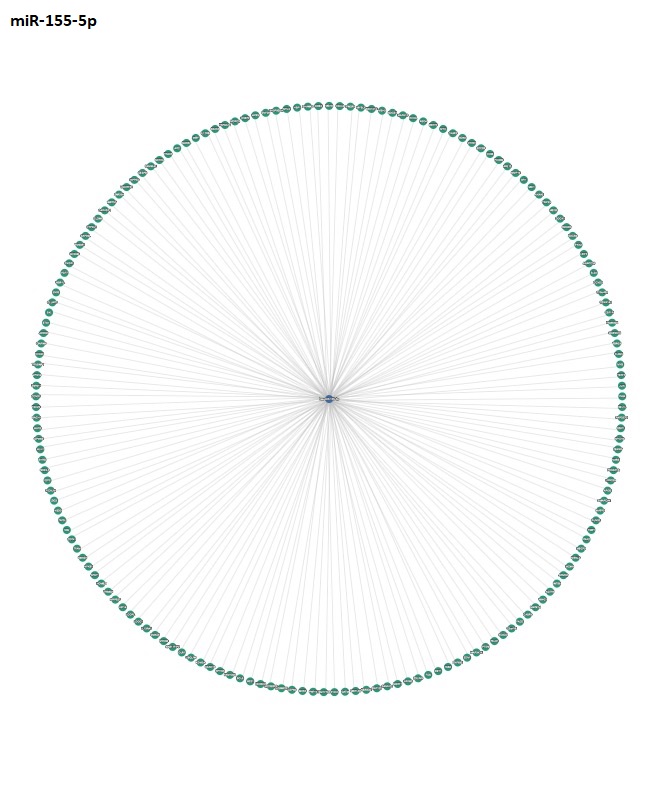

Supplement: Supplementary file 9 [file R1_Appendix_9.jpg]
