# Supplementary material for: Role of miRNAs as biomarkers of COVID-19: a scoping review of the status and future directions for research in this field
Source: Biomark Med. 2021 Nov 17:10.2217/bmm-2021-0348. doi: 10.2217/bmm-2021-0348 (PMC8601154; doi:10.2217/bmm-2021-0348)
Supplement: Supplementary file 5 [file R1_Appendix_4.docx]

Number of studies [reference]

miRNAs	Cells		Animals		Human											Total number of studies which the miRNA was deregulated
					Sample derived from blood									Outros		
					Diagnostic*		Severity**			Mortality***		Recovery****				
	Upregulated	Downregulated	Upregulated	Downregulated	Upregulated	Downregulated	Upregulated	Downregulated	Dysregulated	Upregulated	Downregulated	Upregulated	Downregulated	Upregulated	Downregulated	
miR-10b						1 [36]										1
miR-15a						1 [35]	1 [43]									2
miR-15b-5p				1 [28]			1 [30]									2
miR-16		1 [27]						1 [42]			1 [42]					2
miR-16-2-3p					2 [29, 40]											2
miR-18a-5p						1 [40]										1
miR-18b-5p						1 [40]										1
miR-19b-1						1 [35]										1
miR-19b-2						1 [35]										1
miR-20a-5p						1 [40]										1
**miR-21-5p**					**1 [34]**	**3 [30, 33, 40]**		**1 [30]**								**4**
miR-21a		1 [27]														1
miR-26a-5p															1 [37]	1
miR-27a-3p							1 [42]									1
miR-27b-3p							1 [42]									1
miR-29a		1 [27]			1 [41]							1 [41]				2
miR-29b-2-5p						1 [40]										1
miR-29b-3p															1 [37]	1
miR-29c						1 [35]										1
miR-31-5p								1 [30]								1
miR-32-5p						1 [40]										1
miR-34a-5p															1 [37]	1
miR-66-3p^	1 [24]															1
miR-92a-3p								1 [42]			1 [42]					1
miR-96						2 [35, 40]										2
miR-98-5p									1 [31]		1 [42]					2
miR-99a-5p								1 [30]								1
miR-103		1 [27]														1
miR-103a-2-5p													1 [32]			1
miR-125a		1 [27]														1
miR-125b-5p					1 [40]											1
**miR-126-3p**						**2 [33, 34]**		**1 [43]**								**3**
miR-126-5p								1 [43]								1
miR-130a					1 [38]			1 [38]								1
miR-132-3p											1 [42]					1
miR-140-3p				1 [28]												1
miR-142-3p						1 [30]		1 [30]								1
miR-142-5p					1 [40]											1
**miR-144**						**3 [29, 35, 40]**										**3**
miR-145							1 [43]									1
**miR-146a**						**2 [30, 33]**		**2 [30, 43]**								**3**
miR-146a-3p					1 [41]							1 [41]				1
miR-147-3p^	1 [24]															1
miR-148a	1 [26]															1
miR-148a-3p							1 [42]									1
miR-150-5p								1 [42]								1
miR-151-3p								1 [43]								1
miR-155-3p	1 [25]															1
**miR-155-5p**	**1 [25]**				**3 [34, 38, 41]**			**1 [38]**								**4**
miR-181a-2-3p								1 [30]								1
miR-183-5p						2 [29, 40]										2
miR-192-5p											1 [42]					1
miR-194-1						1 [35]										1
miR-195-5p			1 [28]													1
miR-197	1 [27]															1
miR-199a-3p						1 [40]										1
miR-199a-5p							1 [42]									1
miR-199b-3p						1 [40]										1
miR-200c							1 [43]						1 [32]			2
miR-205							1 [43]									1
miR-208a-3p					1 [34]											1
miR-221-3p			1 [28]													1
miR-223		1 [27]														1
miR-323a-3p											1 [42]					1
miR-335-3p									1 [31]							1
miR-340-5p									1 [31]							1
miR-359-5p^	1 [24]															1
miR-369-3p^	1 [24]															1
miR-378a-3p									1 [31]							1
miR-422a				1 [28]												1
miR-424							1 [43]									1
miR-451a						1 [35]		1 [42]								2
miR-451b						1 [35]										1
miR-486-2						1 [35]										1
miR-486-3p							1 [30]									1
miR-486-5p							1 [30]	1 [42]								2
miR-491-5p							1 [42]									1
miR-499-5p					1 [34]											1
miR-505-5p					1 [40]											1
miR-550-5p							1 [43]									1
miR-590-3p	1 [26]															1
miR-618					2 [29, 40]											2
**miR-627-5p**						**2 [29, 40]**	**1 [43]**									**3**
miR-629							1 [43]									1
miR-663a					1 [35]											1
miR-940		1 [25]														1
miR-1244-1					1 [35]											1
miR-1248					1 [35]											1
miR-1973														1 [39]		1
miR-2115-3p													1 [32]			1
miR-3184					1 [35]											1
miR-3605-3p					1 [30]		1 [30]									1
miR-3609					1 [35]											1
miR-3613-5p						1 [40]										1
miR-3687-2					1 [35]											1
miR-3916					1 [35]											1
miR-4485	1 [25]													1 [39]		2
miR-4521						1 [40]										1
miR-4659a-3p					1 [40]											1
miR-5047					1 [35]											1
miR-5695					1 [40]											1
miR-6501-5p					2 [29, 40]											2
miR-6891					1 [35]											1
miR-7846					1 [35]											1
miR-10399-3p					1 [40]											1
let-7a-5p									1 [31]							1
let-7b^^	1 [27]	1 [27]														1
let-7b-3p					1 [41]							1 [41]				1
let-7b-5p													1 [32]			1
let-7d-5p									1 [31]							1
let-7f-5p									1 [31]							1

^ Virus encoded miRNAs

^^ Upregulated in macrophages from volunteers with cystic fibrosis and downregulated in macrophages from volunteers without cystic fibrosis [SARS-CoV- 2 S1 recombinant protein treated macrophages vs. not treated]

* Patients with COVID-19 vs. healthy volunteers

** Severe/critical COVID-19 vs. mild/moderate COVID-19

*** Nonsurvivors vs. survivors

**** Recovery vs. other disease stages
